# New evidence supports the prophage origin of RcGTA

**DOI:** 10.1128/aem.00434-24

**Published:** 2024-08-27

**Authors:** Yongle Xu, Binbin Liu, Nianzhi Jiao, Jihua Liu, Feng Chen

**Affiliations:** 1 Institute of Marine Science and Technology, Shandong University, Qingdao, China; 2 Qingdao Key Laboratory of Ocean Carbon Sequestration and Negative Emission Technology, Shandong University, Qingdao, China; 3 Institute of Marine and Environmental Technology, University of Maryland Center for Environmental Science, Baltimore, Maryland, USA; University of Nebraska-Lincoln, Lincoln, Nebraska, USA

**Keywords:** prophage, RcGTA, evolutionary origin, gene homology, genomic architecture, virion protein

## Abstract

Gene transfer agents (GTAs) are phage-like entities that package and transfer random host genome fragments between prokaryotes. RcGTA, produced by *Rhodobacter capsulatus*, is hypothesized to originate from a prophage ancestor. Most of the evidence supporting this hypothesis came from the finding of RcGTA-like genes in phages. More than 75% of the RcGTA genes have a phage homolog. However, only a few RcGTA homologs have been identified in a (pro)phage genome, leaving the hypothesis that GTAs evolved from prophages through gene loss with only weak evidence. We herein report the discovery of an inducible prophage (vB_MseS-P1) from a *Mesorhizobium sediminum* strain that contains the largest number (12) of RcGTA homologs found in a phage genome to date. We also identified three putative prophages and two prophage remnants harboring 12–14 RcGTA homologs in a *Methylobacterium nodulans* strain. The protein remote homology detection also revealed more RcGTA homologs from other phages than we previously thought. Moreover, the head–tail gene architecture of these newly discovered prophage-related elements closely resembles that of RcGTA. Furthermore, vB_MseS-P1 virions have structural proteins similar to RcGTA particles. Close phylogenetic relationships between certain prophage genes and RcGTA-like genes in Alphaproteobacteria further support the shared ancestry between RcGTA and prophages. Our findings provide new relatively direct evidence of the origin of RcGTA from a prophage progenitor.

**IMPORTANCE:**

GTAs are important genetic elements in certain groups of bacteria and contribute to the genetic diversification, evolution, and ecological adaptation of bacteria. RcGTA, a common type of GTA, is known to package and transfer random fragments of the bacterial genome to recipient cells. However, the origin of RcGTA is still elusive. It has been hypothesized that RcGTA evolved from a prophage ancestor through gene loss. However, the few RcGTA homologs identified in a (pro)phage genome leave the hypothesis lacking direct evidence. This study uncovers the presence of a large number of RcGTA homologs in an inducible prophage and several putative prophages. The similar head–tail gene architecture and structural protein compositions of these newly discovered prophage-related elements and RcGTA further demonstrate an unprecedentedly observed close evolutionary relationship between prophages and RcGTA. Together, our findings provide more direct evidence supporting the origin of RcGTA from prophage.

## INTRODUCTION

The acquisition of new genetic features via horizontal gene transfer is important for the evolutionary adaptation of prokaryotes. Although different mechanisms facilitating the exchange of genetic materials (e.g., transformation, transduction, and conjugation) have been thoroughly studied in prokaryotes (1–3), another mechanism mediated by gene transfer agents (GTAs) appears to be less understood (4). GTAs are phage-like entities that are produced by a wide range of bacterial and archaeal species (4). GTA genes are part of prokaryotic genomes and can be conserved in certain groups of prokaryotes. When GTA genes are expressed, GTA particles randomly package genome fragments of the producing cell and transfer them to recipient cells through a transduction-like mechanism (4–6). To date, five types of genetically distinct GTAs have been identified, of which RcGTA (7) and BaGTA (8) are produced by Alphaproteobacteria (*Rhodobacter capsulatus* and *Bartonella* spp.), whereas Dd1 (9), VSH-1 (10), and VTA (11) are produced by Deltaproteobacteria (*Desulfovibrio desulfuricans*), Spirochaete (*Brachyspira hyodysenteriae*), and Archaea (*Methanococcus voltae*), respectively (4). Even though GTAs are part of host genomes, they are generally not inducible like prophages. An exception is the VSH-1 GTA, which can be induced by DNA-damaging agents and may cause cell lysis during particle release (4, 10, 12).

One of the most comprehensively studied GTAs is RcGTA, which was first discovered in 1974 (13). The RcGTA particles resemble a siphovirus (14). Its “genome” contains at least 24 genes that are dispersed at five loci on the *R. capsulatus* chromosome (15). A cluster of 17 genes in a single locus encodes proteins mainly responsible for the RcGTA head and tail morphogenesis and are usually mentioned as the head–tail gene cluster (4). The other seven genes in the other four loci are responsible for the production, release, and cell recognition and binding of RcGTA (15).

In addition to *R. capsulatus*, RcGTA-like genomes have also been found in other Alphaproteobacteria species, including those belonging to the orders Rhizobiales, Rhodobacterales, Caulobacterales, Hyphomonadales, Maricaulales, Parvularculales, and Sphingomonadales (16). The RcGTA-like head–tail cluster genes are thought to be more conserved than the other RcGTA-like genes (15). A total of 91 RcGTA-like head–tail clusters were identified in alphaproteobacterial genomes by (16), with each containing at least nine RcGTA homologs. Notably, five RcGTA-like head–tail clusters (i.e., LC1–LC5) were detected in the genome of *Methylobacterium nodulans* ORS 2060 (16). In addition, RcGTA-like head–tail gene clusters have also been detected in *Pseudomonas bauzanensis* W13Z2 (Gammaproteobacteria) and *Streptomyces purpurogeneiscleroticus* NRRL B-2952 (Actinobacteria) (16).

RcGTA has been inferred to originate from a prophage ancestor that subsequently lost the lysogeny-related and DNA replication-related genes as well as most of the regulatory genes in an alphaproteobacterial progenitor that gave rise to the aforementioned five bacterial orders containing RcGTA-like genomes (4, 16). This hypothesis was mainly supported by the finding of a certain number of RcGTA homologs related to structural and regulatory genes in phages (17). The RcGTA homologs were first identified in several temperate phages, such as *Escherichia* phage HK97 and *Streptomyces* phage phiC31, which have a prophage lifestyle and possess genes that are similar to the portal, prohead protease, and major capsid protein genes of RcGTA (18). Homologs of the RcGTA tail-related genes were then identified in several lytic phages infecting members of the order Rhodobacterales (19–24). Us of the RcGTA homologs to screen 1,783 phage genomes revealed that 18 of the 24 RcGTA genes have phage homologs (16). Moreover, the RcGTA-like head–tail cluster has a genomic architecture resembling that of a siphovirus (17). Furthermore, 13 of 17 RcGTA head–tail cluster genes have homologs in phage genomes (16). However, relatively few phages contain a substantial number of RcGTA gene homologs. Temperate phage 16–3 of *Rhizobium meliloti* 41 reportedly contains the most RcGTA homologs (16, 25). Eight RcGTA gene homologs were detected in the *Rhizobium* phage 16–3 genome (16). The homology between RcGTA and phage genes provides indirect evidence that RcGTA may have originated from a prophage. However, the discovery of prophages that share genomic features with RcGTA and contain most of the RcGTA gene homologs may provide more direct evidence.

In this study, we identified an inducible prophage from an Alphaproteobacterium, *Mesorhizobium sediminum* CBW1107-2, as well as three putative prophages and two prophage remnant segments in another alphaproteobacterial (*M. nodulans* ORS 2060) genome, which all possess numerous RcGTA homologs and a similar genomic architecture of the structural genes to that of RcGTA. The identification of these new prophage-associated RcGTA elements provides further evidence that RcGTA originated from prophage ancestors via genome reduction.

## RESULTS AND DISCUSSION

### Identification of an inducible prophage with numerous RcGTA gene homologs

**Fig 1:**
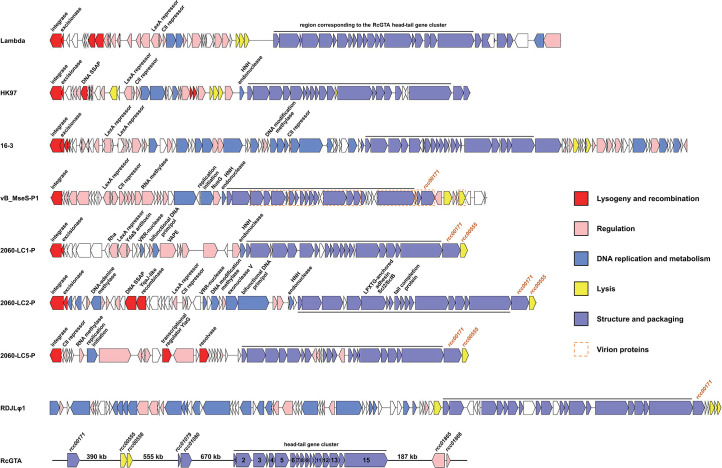
Genomic organization of *Mesorhizobium* phage vB_MseS-P1 and the *M. nodulans* ORS 2060 putative prophages associated with LC1, LC2, and LC5 as defined by Shakya *et al*. (16). The genomes of typical phages (*Escherichia* phage lambda, *Escherichia* phage HK97, *Rhizobium* phage 16–3, and *Roseobacter* phage RDJLφ1) and RcGTA were used as references. The genomic organizations for *Escherichia* phage lambda, *Escherichia* phage HK97, *Rhizobium* phage 16–3, and *M. nodulans* ORS 2060 putative prophages associated with LC2 and LC5 are shown, starting with the integrase gene on the left for ease of comparison. Genes with distinct functions are indicated by different colors. The virion proteins of vB_MseS-P1 detected by mass spectrometry analyses are indicated by orange dashed frames. The RcGTA ORFs in the head–tail gene cluster were numbered with g1–15. The genome regions of each phage corresponding to the RcGTA head–tail gene cluster are marked with lines above or below the ORF bars. Prophage hallmark genes, including integrase, excisionase, LexA repressor, and CII repressor genes, are shown with annotations in the genome of each temperate phage that has a lysogeny life style. Except ORFs corresponding to the RcGTA head–tail cluster genes, the *M. nodulans* ORS 2060 putative prophage (2060-LC1P, 2060-LC2P, and 2060-LC5P) ORFs with homologs in the POG database are exhibited with annotations. Other phage ORFs that are homologous to the *M. nodulans* ORS 2060 putative prophage ORFs outside of the head–tail gene cluster were also shown with annotations. The RcGTA gene homologs excluding those corresponding to head–tail cluster genes in each phage genome are shown with annotations in orange font. DNA prim/pol, DNA primase/polymerase; DNA SSAP, DNA single-strand annealing protein; VRR-nuclease, virus-type replication repair nuclease; VAPE, virulence-associated protein E.

A temperate siphovirus, vB_MseS-P1 (Fig. S1), was induced from the Alphaproteobacterium *M. sediminum* CBW1107-2 using mitomycin C. The host CBW1107-2 was isolated from an estuarine *Synechococcus* culture and shares a 100% 16S rRNA gene sequence similarity with the type strain of *M. sediminum*, YIM M12096^T^, which was isolated from a deep-sea sediment sample from the Indian Ocean (26). *Mesorhizobium* species belong to the order Rhizobiales and are usually found in the rhizosphere (27), but they have also been isolated from marine, freshwater, and phycosphere microbiota (26, 28–31). vB_MseS-P1 has a double-stranded DNA genome with a length of 40,795 bp and a G + C content of 63.3%; its integration in the host genome has been observed (GenBank accession no. JAZHFV000000000). A total of 63 open reading frames (ORFs) were predicted in the vB_MseS-P1 genome, with 43 ORFs predicted to be related to lysogeny and recombination, regulation, DNA replication, and structure formation (Fig. 1; Tables S1 and S2). All vB_MseS-P1 ORFs showed the highest amino acid sequence identity with proteins of a bacterial origin in the non-redundant (NR) database (Table S1), indicative of a greater similarity to prophage elements than to lytic phages. Notably, vB_MseS-P1 shares nine homologous genes with the alphaproteobacterial RcGTA-like elements, including eight homologs of the head–tail cluster genes and one homolog of *rcc00171*, which encodes a tail fiber protein (4) (Table S3). Thus, vB_MseS-P1 replaces *Rhizobium* phage 16–3 (16) as the phage that contains the most RcGTA homologs.

Homolog detection between vB_MseS-P1 and the 91 RcGTA-like head–tail clusters in alphaproteobacterial genomes revealed that vB_MseS-P1 shares more homologous genes (i.e., seven) with *M. nodulans* ORS 2060 gene clusters LC2 and LC3 than with the other alphaproteobacterial head–tail clusters (i.e., less than five) (Table S3). Similar to vB_MseS-P1, *Rhizobium* phage 16–3 also shares more homologs with *M. nodulans* ORS 2060 LC2 and LC3 than with the other alphaproteobacterial RcGTA-like head–tail clusters. However, vB_MseS-P1 and *Rhizobium* phage 16–3 differ in terms of the specific homologs they share with *M. nodulans* ORS 2060 LC2 and LC3 as well as the other alphaproteobacterial head–tail clusters. Specifically, vB_MseS-P1 shares homologs of RcGTA g3, 4, 7, 8, 9, 10, and 11 with *M. nodulans* ORS 2060 LC2 and LC3 as well as homologs of RcGTA g3, 4, 6, 8, and 9 with other alphaproteobacterial head–tail clusters. In contrast, *Rhizobium* phage 16–3 shares homologs of RcGTA g2, 3, 7, 8, 9, 10, and 11 with *M. nodulans* ORS 2060 LC2 and LC3 as well as homologs of RcGTA g3, 4, 5, 6, and 8 with the other alphaproteobacterial head–tail clusters (Table S3).

### *Methylobacterium nodulans* ORS 2060 gene clusters LC1–LC5 belong to putative prophages or prophage remnants

The difference between *M. nodulans* ORS 2060 LCs and other alphaproteobacterial RcGTA-like head–tail clusters was also revealed in a previous study. As demonstrated by Shakya *et al*. (16), the phylogeny of the 91 alphaproteobacterial head–tail clusters is almost congruent with that of a set of conserved genes belonging to the corresponding Alphaproteobacteria at the order and family levels. Remarkably, in the head–tail cluster phylogeny, the five *M. nodulans* ORS 2060 head–tail clusters (LC1–LC5) were grouped outside of Rhizobiales (i.e., the order that *M. nodulans* ORS 2060 belongs to), which is inconsistent with the conserved gene reference tree (16).

The viral homologous genes within or flanking the *M. nodulans* ORS 2060 LCs also indicate that these five LCs differ from the routine RcGTA-like gene clusters and are associated with prophages. The genes flanking the *M. nodulans* ORS 2060 head–tail clusters LC1–LC5 were re-examined to verify whether they are phage-related genes and whether the five head–tail clusters are parts of prophages. An integrase gene was found at the 27th ORF upstream of LC1 as well as at the 38th and 24th ORF downstream of LC2 and LC5, respectively, in the genome of *M. nodulans* ORS 2060. Moreover, ORFs corresponding to excisionase, LexA repressor, and CII repressor genes (i.e., prophage hallmark genes) were also identified within the regions from the integrase genes to LC1, LC2, and LC5, respectively. Sequences that started with the integrase gene and ended with the lysis gene in LC1, LC2, and LC5 were used to screen for homologs in the Phage Orthologous Group (POG) database. In addition to ORFs homologous to RcGTA-like head–tail cluster genes, 10, 13, and eight additional ORFs from the LC1-, LC2-, and LC5-associated prophage-like segments, respectively, were assigned to POGs and designated as viral genes (Fig. 1; Table S4). The detected homologs and the loci of specific genes also revealed similarities between the *M. nodulans* ORS 2060 prophage-like segments and specific phages with a lysogenic lifestyle. For example, in addition to the RcGTA-like homologous ORFs, seven, eight, and three ORFs from the LC1-, LC2-, and LC5-associated prophage-like segments were homologous to the predicted ORFs in *Rhizobium* phage 16–3, vB_MseS-P1, and HK97. Additionally, the locus comprising an HNH endonuclease gene adjacent to the head–tail cluster in the LC1 and LC2 prophage-like segments resembled the organization of the corresponding ORFs in *Rhizobium* phage 16–3, vB_MseS-P1, and HK97 (Fig. 1). Moreover, since phage integration sites often occur in tRNA genes (32), the tRNA genes immediately adjacent to the integrase genes associated with LC1, LC2, and LC5 further increase the possibility that LC1, LC2, and LC5 belong to prophage segments rather than RcGTA-like regions.

No integrase genes were detected flanking LC3 and LC4 in the genome of *M. nodulans* ORS 2060. Aside from the ORFs homologous to RcGTA-like head–tail elements, six and five additional ORFs within LC3 and LC4, respectively, and their respective ten flanking ORFs upstream and downstream were assigned to specific POGs and designated as viral genes (Table S4). Furthermore, the amino acid sequences encoded by many LC3 ORFs were highly similar to the sequences encoded by the predicted ORFs in LC1, LC2, and LC5; the similarities were highest for the ORFs in LC2 (sequence identity ranging from 93.2% to 100%). Seven LC4 ORFs related to the tail and baseplate were homologous to ORFs in LC1, LC2, LC5, and LC3, with the encoded amino acid sequence identities exceeding 90% for the ORFs corresponding to the distal tail (95.3%–98.1%), hub (94.2%–100%), peptidase (94.7%–100%), and megatron (95%–99.8%) proteins. The numerous homologs between the LC3- and LC4-associated segments and the putative prophage segments containing LC1, LC2, and LC5 with high encoded amino acid sequence identities suggest LC3 and LC4 may be prophage remnants.

### An unprecedentedly large number of RcGTA gene homologs were identified in the *M. nodulans* ORS 2060 LC1–LC5-associated prophage segments

**Fig 2:**
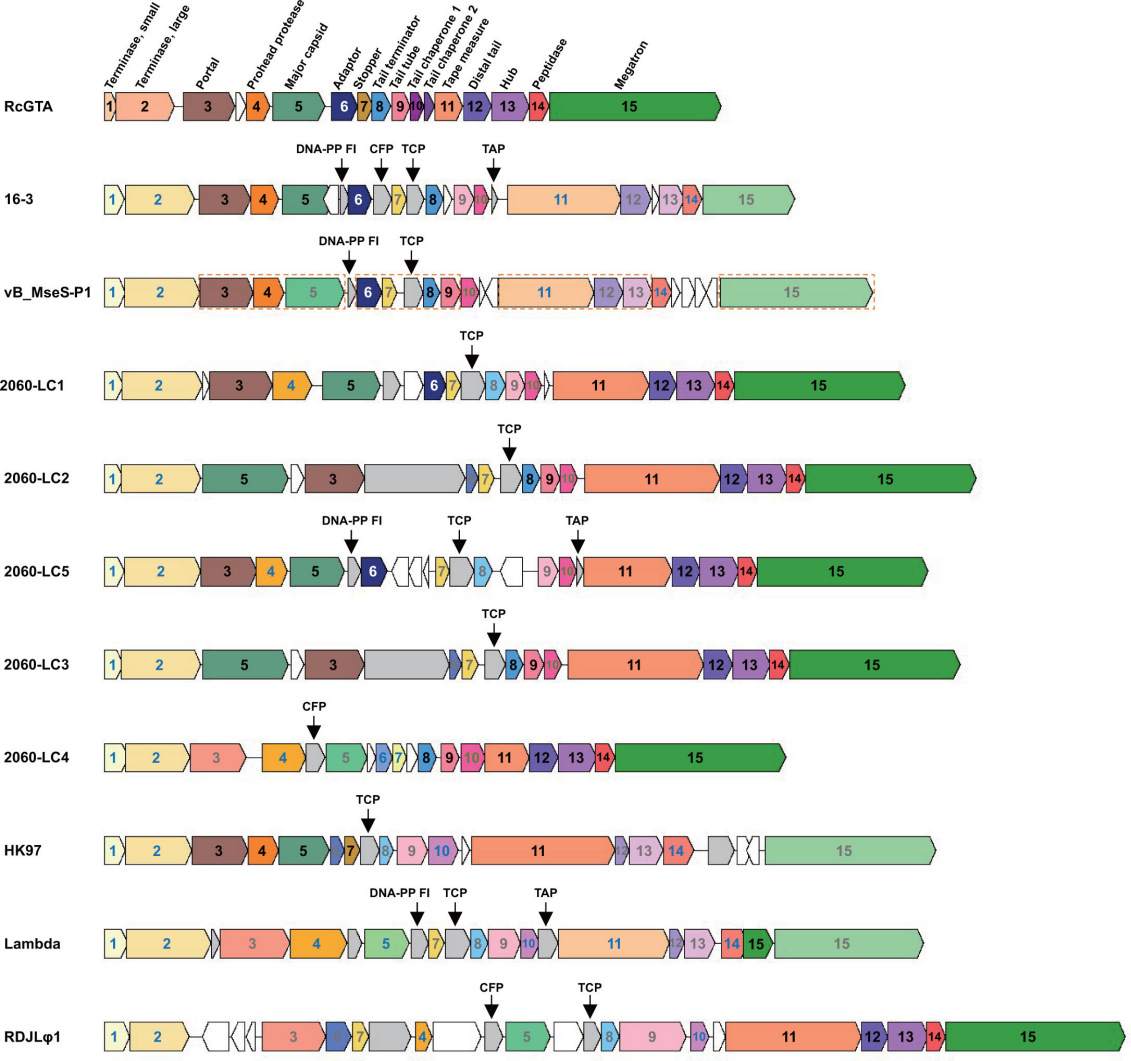
Comparison of the RcGTA head–tail gene cluster and its corresponding structural gene regions in the genomes of four typical temperate phages and the *M. nodulans* ORS 2060 LC1–LC5. The structural gene region of RDJLφ1 was used as a reference. Both LC2 and LC5 of *M. nodulans* ORS 2060 are shown starting with the terminase small subunit gene on the left for ease of comparison. The numbering of the ORFs in the RcGTA head–tail gene cluster are the same with that of the RcGTA head–tail gene cluster in Fig. 1. The phage ORFs encoding proteins with the same predicted functions as the proteins encoded by the RcGTA genes are marked with the same numbers as their RcGTA counterparts. The phage ORF numbers are colored as follows: black or white (for g6), shares amino acid sequence identities with the corresponding genes in RcGTA-like elements; green, shares the same conserved domain with RcGTA g10; gray, shares protein structural identities with the RcGTA proteins determined by cryo-electron microscopy (33); blue, has the same predicted functions as the corresponding RcGTA proteins without sequence-, conserved domain-, and structure-based similarity. The RcGTA head–tail cluster genes are indicated by different colors, while the corresponding genes with the same functions in the phage segments are presented in the same or lighter colors according to their encoded amino acid sequence or structural identities with the RcGTA-like proteins. The ORFs in gray bars indicate structural genes other than the head–tail cluster genes in the phage segments, whereas the ORFs in white bars indicate genes encoding nonstructural proteins. The ORFs encoding the virion proteins of vB_MseS-P1 detected by mass spectrometry are indicated by orange dashed frames. TCP, tail completion protein; TAP, tail assembly protein; DNA-PP FI, DNA-packaging protein FI; CFP, capsid fiber protein.

The *M. nodulans* ORS 2060 gene clusters LC1–LC5 all contain a substantial number of homologs of the RcGTA-like head–tail cluster genes identified in Alphaproteobacteria. Specifically, LC2 and LC3 both share nine homologs of g3, 5, 8, 9, and 11–15 with the other alphaproteobacterial RcGTA-like head–tail clusters. Both LC1 and LC5 share eight homologs of g3, 5, 6, and 11–15, whereas LC4 shares seven homologs of g8, 9, and 11–15 with the other alphaproteobacterial RcGTA-like head–tail clusters (Fig. 2; Table S5). Most of the RcGTA homologs in LC1–LC5 show the highest sequence identities with the RcGTA-like elements from the members of the order *Rhizobiales* (Table S5), which includes *M. nodulans* ORS 2060. In addition, the *M. nodulans* ORS 2060 gene clusters LC1–LC5-associated prophage segments also harbor two RcGTA homologs of *rcc00171* and *rcc00555* (Fig. 1). These homologs are located adjacent to the head–tail cluster and share amino acid identities of 45.2%–46.6% with *rcc00171* and 37.8%–44.9% with *rcc00555*. Notably, the encoded amino acid sequence identities between *rcc00171* and the *M. nodulans* ORS 2060 gene clusters LC1–LC5-associated homologs are much higher than those between *rcc00171* and other phage homologs, including those of RDJLφ1 (33.3%), vB_MseS-P1 (29.9%), and vB_DshS-R4C (34.1%). Since the RcGTA-like gene homolog determination from *Rhizobium* phage 16–3 and vB_MseS-P1 was partially based on the homology between the phage genes and the *M. nodulans* ORS 2060 LC1–LC5 genes, the accurate numbers of RcGTA-like gene homologs from *Rhizobium* phage 16–3 and vB_MseS-P1 should be five and six, respectively. Therefore, among (pro)phage-associated genome sequences, LC2 and LC3 contain the most RcGTA-like gene homologs (Table 1).

**TABLE 1:** Numbers of RcGTA-like gene homologs identified in each phage or prophage genome using different methods

Phage or putative prophage	Sequence-based homolog no.	Structure-based homolog no.^*a*^	Rcc01692 CDD^*b*^	Total homolog no.
*Rhizobium* phage 16–3	5	9	*+*	11
*Mesorhizobium* phage vB_MseS-P1	6	9	*+*	12
*M. nodulans* ORS 2060 LC1-related putative prophage	10	9	*+*	14
*M. nodulans* ORS 2060 LC2-related putative prophage	11	9	*+*	14
*M. nodulans* ORS 2060 LC5-related putative prophage	10	9	*+*	14
*M. nodulans* ORS 2060 LC3-related prophage remnants	11	9	*+*	14
*M. nodulans* ORS 2060 LC4-related prophage remnants	9	7	*+*	12
*Escherichia* phage HK97	5	9	*-*	11
*Escherichia* phage Lambda	1	7	*-*	7
*Roseobacter* phage RDJLφ1	6	9	*-*	12

^*a*^ Phage homologs of the nine structurally characterized RcGTA proteins encoded by genes in the head–tail cluster (g3, 5, 6, 7, 8, 9, 12, 13, and 15) (33).

^*b*^ Presence of a gene sharing the conserved domain pfam11836 with Rcc1692 that is encoded by the RcGTA g10.

### Remote homology detection revealed more phage homologs of the RcGTA elements

Although the *M. nodulans* ORS 2060 LC1–LC5, vB_MseS-P1, and *Rhizobium* phage 16–3 genes were not homologous to RcGTA tail chaperone 1 gene (g10) according to amino acid sequence analyses, these phages all possess a gene that shares the conserved domain pfam11836 with RcGTA g10, suggesting it may be a distant homolog of RcGTA g10. As protein structures change much slower than sequences, structural similarities may reflect the relationships between proteins (34). Therefore, as an alternative to sequence searches to deduce protein homology, estimating protein structure similarities is useful for inferring remote homology between proteins (35). Nine RcGTA proteins encoded by genes in the head–tail cluster (g3, 5, 6, 7, 8, 9, 12, 13, and 15) were previously structurally characterized via cryo-electron microscopy determination and reconstruction of the RcGTA particles (33). Deconstructing the RcGTA particle proteins enables the detection of remote homology between phage and RcGTA structural proteins. In addition to the homologs determined according to sequence analyses, LC2 and LC3 were both predicted to include genes encoding two additional proteins that are structurally homologous to the proteins encoded by RcGTA g6 and 7 through the HHpred remote protein homology search (35). Additionally, LC1 and LC5 were predicted to include genes encoding three additional proteins that are structurally homologous to the proteins encoded by RcGTA g7, 8, and 9, whereas LC4 includes genes encoding two additional proteins that are structurally homologous to the proteins encoded by RcGTA g3 and 5 according to their predicted protein structural properties (Fig. 2; Table S4).

Moreover, although sequence analyses indicated only five structural proteins are homologous to the RcGTA-like head–tail proteins, vB_MseS-P1 and *Rhizobium* phage 16–3 both encode proteins that are homologous to the nine RcGTA structural proteins according to the predicted protein structural properties (Fig. 2; Table S2). Furthermore, viral genomes encoding multiple proteins with predicted structural homology to the RcGTA proteins are not rare (Table 1). The genomes of siphophages HK97 and RDJLφ1 also encode all nine RcGTA protein homologs, whereas the Lambda phage genome only lacks the homologs of RcGTA g5 and g6 according to their predicted protein structural analyses (Fig. 2; Table S2). It is likely that as more RcGTA protein structures are determined, homology between phage and RcGTA proteins will be further discovered. The substantial number of RcGTA protein homologs in the *M. nodulans* ORS 2060 LC1–LC5 and the aforementioned phage genomes further indicates the close evolutionary relationship between the RcGTA-like elements and phages.

### Similarity in the architecture of the head–tail genes between phages and RcGTA

In terms of their organization, the genomic regions encoding structural proteins in *M. nodulans* ORS 2060 LC1–LC5, vB_MseS-P1, *Rhizobium* phage 16–3, and HK97 are highly similar to the RcGTA head–tail gene cluster. The arrangements of the phage genes corresponding to RcGTA g1–5 and g11–15 are relatively conserved (Fig. 2). The g1–5 and g11–15 clusters in LC5 are both arranged consecutively. The structural gene regions of vB_MseS-P1, *Rhizobium* phage 16–3, and HK97 consist of a consecutively arranged g1–5 gene cluster and a variable g11–15 gene cluster with uncertain insertions in each genome. In contrast, LC1–LC4 and phage Lambda contain a variable g1–5 cluster and a consecutively arranged g11–15 cluster (Fig. 2). Additionally, LC2–LC4 only contain three genes that are unrelated to the RcGTA head–tail cluster. However, LC2 and LC3 do not encode a typical major capsid protein and a prohead protease, but they have a longer major capsid protein gene containing an additional prohead protease domain. Moreover, the longer major capsid protein gene (g5) in LC2 and LC3 is located ahead of the portal protein gene (g3) and an additional unknown gene. The architecture of the phage region counterparts from g6 to g10 is more variable, with specific genes inserted at certain loci in different phage-associated genomes (Fig. 2). In the lytic phage RDJLφ1, the g11–15 cluster is arranged consecutively, whereas numerous genes are inserted in the g1–10 cluster, and the g6–7 gene cluster is positioned ahead of g4–5, which differs from the arrangement in the phages with a lysogenic lifestyle analyzed in this study (Fig. 2). In addition, the consecutive tail genes seem to be more conserved. The sequence similarities between the tail genes of *M. nodulans* ORS 2060 LC1–LC5, RDJLφ1, and the RcGTA-like elements are much higher than those between the other genes (Tables S3 and S5).

### Phage homologs other than structural genes within the RcGTA-like head–tail clusters

Intriguingly, certain genes predicted within the RcGTA-like head–tail cluster regions of alphaproteobacterial genomes are not associated with the 17 RcGTA homologs, but have some homology with genes in phages that harbor multiple RcGTA homologs. Notably, a DNA-cytosine methylase gene located within the RcGTA-like head–tail cluster region of *Rhodopseudomonas palustris* BisB5 (ABE39207.1) and *Rhodopseudomonas palustris* DX-1 (ADU45192.1) is homologous to ORF25 predicted in vB_MseS-P1. A lysozyme gene positioned between the terminase large subunit and portal protein genes of the RcGTA-like head–tail clusters in the genomes of *Agrobacterium* sp. H13-3 (ADY63872.1), *Agrobacterium fabrum* 1D132 (AYM56651.1), *Agrobacterium vitis* S4 (ACM35950.1), *Agrobacterium fabrum* C58 (AAK86758.2), and *Rhizobium* sp. IRBG74 (CDI07854.1) is homologous to ORF29 (YP_002117587.1) predicted in *Rhizobium* phage 16–3. The presence of phage gene homologs other than the RcGTA structural homologs within the RcGTA-like elements further suggests a close evolutionary relationship between the RcGTA-like elements and phages. It can be inferred that vB_MseS-P1 and *Rhizobium* phage 16–3 are likely the closest genuine phage relatives of these RcGTA-like elements.

### Close phylogenetic relationships between phage and RcGTA-like genes

**Fig 3:**
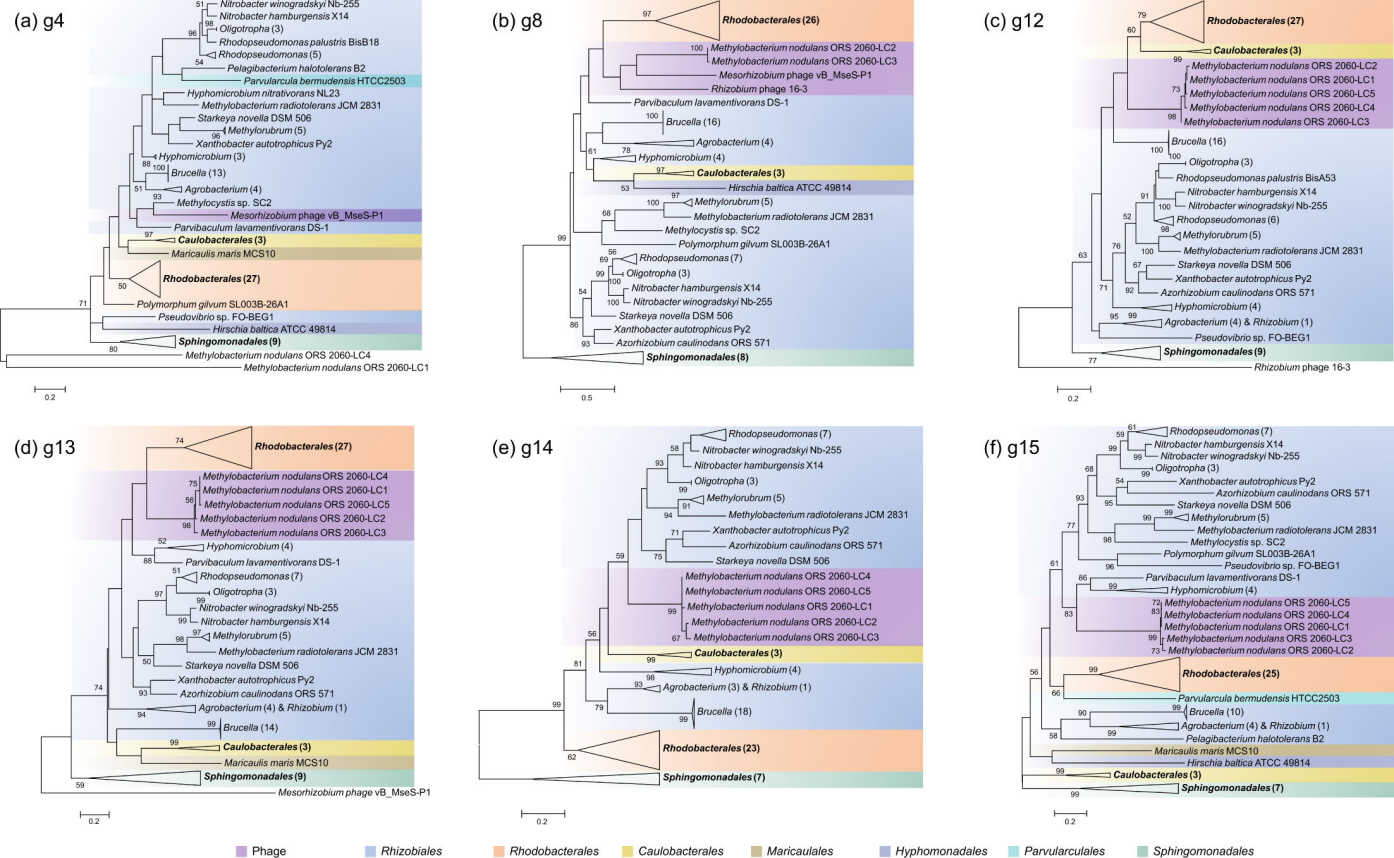
Phylogenetic relationships between specific phage genes and their RcGTA-like homologs found in alphaproteobacteria. Maximum-likelihood phylogenetic trees were constructed based on the amino acid sequences with a bootstrap replicate of 100. In the trees of the head–tail cluster genes g4 (**a**), g12 (**c**), and g13 (**d**), gene counterparts in *M. nodulans* ORS 2060 LC4 and LC1, *Rhizobium* phage 16–3, and vB_MseS-P1 were used as the outgroup, respectively. Sequences that form very deep branches were excluded in trees of g8, g12, g13, and g14 to obtain a more stable topology structure. The numbers of sequences for each compressed cluster are included in parentheses. Bootstrap values of >50% are shown near each node.

The phylogenetic relationships between specific phage genes and their counterparts in the alphaproteobacterial RcGTA-like head–tail clusters were investigated. Among the RcGTA homologs in vB_MseS-P1, 16–3, and *M. nodulans* ORS 2060 LC1–LC5, numerous genes had close phylogenetic relationships with their counterparts in alphaproteobacterial RcGTA-like head–tail clusters (Fig. 3). The g4 (*rcc01686*) homolog in vB_MseS-P1 clustered together with their RcGTA-like counterparts was identified in all seven alphaproteobacterial orders (Fig. 3). The g8 (*rcc01690*) homologs in vB_MseS-P1, *Rhizobium* phage 16–3, and *M. nodulans* ORS 2060 LC2 and LC3 as well as the g12 (*rcc01695*), g13 (*rcc01696*), and g14 (*rcc01697*) homologs in *M. nodulans* ORS 2060 LC1–LC5 clustered with their RcGTA-like counterparts were identified in orders of Rhizobiales, Rhodobacterales, and Caulobacterales, respectively. Notably, the phylogenetic relationships between the phage genes and their RcGTA-like g8, g12, g13, and g14 homologs identified in Rhizobiales, Rhodobacterales, and Caulobacterales are closer than those between genes identified in Sphingomonadales and Rhizobiales, Rhodobacterales, and Caulobacterales (Fig. 3b through e). The g15 (*rcc01698*) homolog in in *M. nodulans* ORS 2060 LC1–LC5 clustered together with their RcGTA-like counterparts was identified in orders of Rhizobiales, Rhodobacterales, and Parvularculales and show closer relationships with these three orders than those identified in the other four alphaproteobacterial orders (Fig. 3f). The close phylogenetic relationships between specific viral genes and the alphaproteobacterial RcGTA-like genes imply that they may evolve from the same origin.

### Additional structural genes in the phage genome region corresponding to the RcGTA head–tail cluster

Compared with RcGTA, phages usually contain more structural genes. In the region corresponding to the RcGTA head–tail cluster, the phage or prophage-associated genomes examined in this study contained an additional tail completion protein (TCP) gene located immediately upstream of the tail terminator protein gene, with the exception of the *M. nodulans* ORS 2060 LC4 segment. Furthermore, the TCP genes in vB_MseS-P1, *Rhizobium* phage 16–3, HK97, and *M. nodulans* ORS 2060 LC1–LC3 and LC5 belong to the HK97 gp10 family, indicating they are conserved within phage genomes and important for phage structure formation. Additionally, a DNA packaging protein FI gene was identified downstream of the major capsid protein gene in the genomes of vB_MseS-P1, *Rhizobium* phage 16–3, *M. nodulans* ORS 2060 LC5, and phage Lambda. A tail assembly protein gene was detected downstream of the tail assembly chaperone gene in the genome of *Rhizobium* phage 16–3, *M. nodulans* ORS 2060 LC5, and phage Lambda; this gene may contribute to virion assembly in a manner similar to that of the RcGTA tail chaperone 2 gene (g10.5). Moreover, a capsid fiber protein gene, which may have a function similar to that of the RcGTA head fiber protein encoded by a gene outside of the head–tail cluster, was detected within the structural region of *Rhizobium* phage 16–3, RDJLφ1, and *M. nodulans* ORS 2060 LC4 (Fig. 2). The absence or divergence of phage genes within the RcGTA head–tail cluster can be attributed to the gene loss, rearrangement, exchange, or nucleotide base substitutions that occurred after the phage ancestor of RcGTA resided in the host genome or to the acquisition of genes by phage genomes during evolution.

### Similarity in the structural protein compositions of vB_MseS-P1 virions and RcGTA particles

A mass spectrometry analysis identified 15 virion proteins encoded in the vB_MseS-P1 genome. Among these 15 proteins, 11 were encoded by structural ORFs corresponding to those encoding the RcGTA head–tail cluster proteins, including g3–9, g11–13, and g15, nine of which were detected in RcGTA particles via cryo-electron microscopy analyses (33). In addition, the tail fiber protein of vB_MseS-P1, which is homologous to Rcc00171, was also detected in the virion (Fig. 1 and 2). The presence of proteins with similar functions suggests that the structure of the vB_MseS-P1 virions closely resembles that of RcGTA particles. Furthermore, TCP and a lysozyme protein were also detected in the vB_MseS-P1 virions, representative of the difference between genuine phage virions and RcGTA particles. More thorough analyses of virion proteomes may help further reveal the virion structural similarities between phages and RcGTA.

### Conclusions

This study identified an inducible prophage (vB_MseS-P1) and five new prophage-related segments in the *M. nodulans* ORS 2060 genome. An unprecedentedly large number of RcGTA-like homologs were detected in the genome of vB_MseS-P1 and the five *M. nodulans* ORS 2060 prophage-associated segments. In addition, the head–tail gene architecture of these newly discovered prophage-related elements closely resembles that of RcGTA. Close phylogenetic relationships were observed between several of the prophage genes and their RcGTA homologs. Moreover, vB_MseS-P1 virions and RcGTA particles have similar structural protein compositions. The finding that prophages share a large number of RcGTA-like homologs reflects an unprecedentedly observed close evolutionary relationship between prophages and RcGTA-like elements. Furthermore, vB_MseS-P1 and the *M. nodulans* ORS 2060 putative prophages may represent the closest phage relatives of the putative prophage ancestor that gave rise to RcGTA, thereby providing new forceful evidence for the hypothesis that GTAs originated from prophages. Notably, the hosts of all prophage-related elements that possess numerous RcGTA-like homologs in this study (i.e., vB_MseS-P1, 16–3, and *M. nodulans* ORS 2060 LC1–5) belong to the order Rhizobiales. Further comparative analyses of these and other phages harboring multiple RcGTA homologs will continue to provide insights into the evolutionary trajectories connecting phages with GTAs.

## MATERIALS AND METHODS

### Prophage induction and amplification

The *M. sediminum* CBW1107-2 bacterial strain isolated from the *Synechococcus* sp. CBW1107 culture was grown in rich organic (RO) medium (36). For the prophage induction, mitomycin C (final concentration of 0.5 mg L^−1^) was added to 1 L cultures of exponentially growing bacteria (OD_600_ = 0.2). After a 30-minute treatment with mitomycin C, the bacterial cells were pelleted and washed twice with fresh RO medium by centrifugation at 6,000 × g for 10 minutes. The resuspended cultures in fresh RO medium were incubated at 28°C with shaking (160 rpm) for 24–48 hours. After the cells were lysed, phage particles were harvested and purified as described by Xu *et al*. (20), with some modifications. Briefly, phage lysates were treated with DNase I and RNase A (both at 2 mg L^−1^) at room temperature for 1 hour, after which the NaCl concentration of the lysates was adjusted to 1 M prior to an incubation on ice for 1 hour. The remaining cells were removed by centrifugation and 0.45-µm filtration. Phage particles in the filtrates were concentrated by polyethylene glycol 8000 precipitation and then purified by CsCl-mediated ultracentrifugation (200,000 × g for 5 hours) using the SW 41Ti Rotor and the Optima L-100XP ultracentrifuge (Beckman Coulter, CA, USA). The visible viral bands were extracted and desalted via centrifugal ultrafiltration (30 kDa cutoff). Purified phage suspensions were stored at 4°C in darkness.

### Morphological examination

One drop of the CsCl-purified phage suspension was adsorbed to Formvar/carbon-coated 200 mesh copper grids for 10 minutes and then negatively stained with 0.5% (wt/vol) aqueous uranyl acetate for 30 seconds in darkness. After drying for 2 hours, the grid was examined using the Tecnai G2 Spirit BioTwin transmission electron microscope (FEI Thermo Fisher Scientific, Eindhoven, The Netherlands). Images were captured using a CCD image transmission system (Gatan Inc.).

### Phage DNA extraction, genome sequencing, and assembly

Phage DNA was extracted from the CsCl-purified phage suspension using a phenol/chloroform method as described by Xu *et al*. (20). The genome sequencing analysis was performed using the Illumina MiSeq system at Shanghai Personal Biotechnology Co., Ltd., Shanghai, China. The library was constructed and sequenced according to the manufacturer’s instructions. The quality of the raw reads was assessed using FastQC, and reads were trimmed using the FASTX-Toolkit. The genome was assembled using clean reads and the IDBA-UD algorithm (37).

### Genome annotation and analysis

The ORFs in the induced prophage (vB_MseS-P1) genome were identified using the GeneMark.hmm 2.0 gene prediction program (38) as well as the RAST server (version 2.0) (39) and MetaGene Annotator (40). The translated ORFs were used as queries for a BLASTP search of the NCBI NR protein database (41), which was conducted to find homologs in microbial organisms. Conserved domains were identified using the NCBI Conserved Domain Database (42) to predict the functions of the ORFs in the genomes of vB_MseS-P1, *Rhizobium* phage 16–3 (GenBank accession no. DQ500118.1), *Escherichia* phage HK97 (GenBank accession no. AF069529.1), *Escherichia* phage Lambda (GenBank accession no. J02459.1), *Roseobacter* phage RDJLφ1 (GenBank accession no. NC_015466.1), and five putative prophages or prophage remnants containing the head–tail clusters (LC1–LC5) in the genome of *M. nodulans* ORS 2060 (GenBank accession no. CP001349.1). For the ORFs related to structure formation or the ORFs whose functions were not predicted after the sequence analyses in each phage genome or putative prophage segments, HHpred analyses were conducted using default parameters to predict the functions on the basis of the predicted structural properties (35). The phylogenetic analyses of specific genes were performed using the MEGA 7.0 software package (43). The maximum likelihood method based on the Jones–Taylor–Thornton (JTT) model with 100 bootstrap replicates was used for phylogenetic tree construction. In the phylogenetic analyses of genes g4, g12, and g13, outgroups were added to obtain a more stable tree topology structure. The outgroup for each tree was selected from the category of vB_MseS-P1, *Rhizobium* phage 16–3, and *M. nodulans* ORS 2060 LC1–LC5, with the sequence yielding the highest bootstrap value at key nodes ultimately chosen as the respective outgroup.

### Virion proteomic determination by mass spectrometry

The CsCl-purified phage suspensions were used for the proteomic analysis of the vB_MseS-P1 virions. Virion proteins were digested according to the FASP procedure described by Wiśniewski *et al*. (44). The resulting tryptic peptides were subjected to liquid chromatography tandem MS analyses using a Q Exactive mass spectrometer (Thermo Fisher Scientific, Waltham, MA) coupled to an Easy nLC 1000 system (Thermo Fisher Scientific) (45). Generated MS/MS spectra were searched against the vB_MseS-P1 genome by using the Mascot2.2 software (Matrix Science, London, UK) to retrieve the data.

### Identification and annotation of putative prophages or prophage remnants associated with the five LCs in the *M. nodulans* ORS 2060 genome

The sequences downstream and upstream of the five LC regions in the *M. nodulans* ORS 2060 genome were screened for the presence of integrase genes. For the sequences that contained a nearby integrase gene, the ORFs starting with the integrase gene and ending with the lysis gene were extracted and reannotated according to the conserved domain and HHpred searches as described previously. For the sequences lacking integrase genes, the ORFs in the LC regions as well as ten ORFs upstream and downstream of the regions were also reannotated using the same methods.

### Detection of the homologs of the *M. nodulans* ORS 2060 putative prophage or remnant prophage ORFs in the POG database and genomes of the four phages with a lysogenic lifestyle included in this study

To examine the similarity of the five *M. nodulans* ORS 2060 putative prophage or remnant prophage segments corresponding to LC1–LC5, defined by Shakya *et al*. (16), to phages, the amino acid sequences encoded by the ORFs in these segments were used as queries for a BLASTP search (E-value <10^−5^; bit score >40) of the POG database (46), which was downloaded (ftp://ftp.ncbi.nlm.nih.gov/pub/kristensen/thousandgenomespogs/blastdb) in November 2022, using NCBI BLAST+ (version 2.7.1). The ORFs were designated as viral if they were assigned to a POG. Homologs of the *M. nodulans* ORS 2060 putative prophage or remnant prophage ORFs were identified in the genomes of the four phages with a lysogenic lifestyle included in this study to determine the similarities with prophages.

### Detection of RcGTA-like head–tail cluster gene homologs in specific phages and putative prophage segments

According to Shakya *et al*. (16), only gene clusters containing at least nine RcGTA homologs represent RcGTA-like elements. Head–tail cluster-related ORFs in the gene clusters retrieved from 86 Alphaproteobacteria, the actinobacterium *Streptomyces purpurogeneiscleroticus* NRRL B-2952, and the Gammaproteobacterium *Pseudomonas bauzanensis* W13Z2, which were identified by Shakya *et al*. (16), and from nine extra Alphaproteobacteria were used to make a database representing a collection of RcGTA-like elements (Table S6). The translated ORFs of vB_MseS-P1, *Rhizobium* phage 16–3, *Escherichia* phage HK97, *Escherichia* phage Lambda, *Roseobacter* phage RDJLφ1, and five putative prophages or prophage remnants containing the head–tail clusters LC1–LC5 in the genome of *M. nodulans* ORS 2060 were used as queries for a BLASTP search (E-value <10^−5^; bit score >40) of the above-mentioned database of RcGTA-like elements.

## Data Availability

The genome sequences of vB_MseS-P1 and *Mesorhizobium sediminum* CBW1107-2 were submitted to the GenBank database under accession no. PP232116 and JAZHFV000000000.

## SUPPLEMENTAL MATERIAL

Supplemental material aem.00434-24-s0001.pdf Fig. S1; Tables S1 to S6.

ASM does not own the copyrights to Supplemental Material that may be linked to, or accessed through, an article. The authors have granted ASM a non-exclusive, world-wide license to publish the Supplemental Material files. Please contact the corresponding author directly for reuse.
