# Supplementary material for: New evidence supports the prophage origin of RcGTA
Source: Appl Environ Microbiol. 2024 Aug 27;90(9):e00434-24. doi: 10.1128/aem.00434-24 (PMC11409702; doi:10.1128/aem.00434-24)
Supplement: Supplemental material — Fig. S1; Tables S1 to S6. [file aem.00434-24-s0001.pdf]

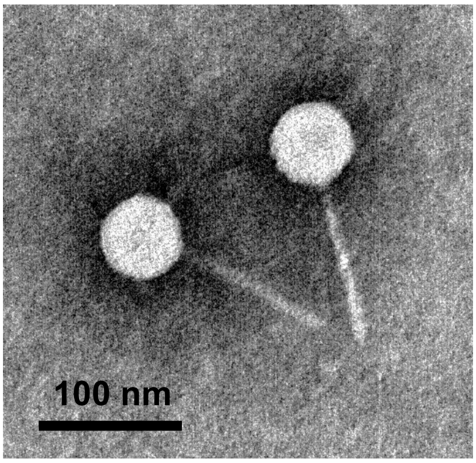

Figure S1. Transmission electron microscopy image of *Mesorhizobium* phage vB_MseS-P1.

Table S1. Predicted ORFs in the vB_MseS-P1 genome with homologs in the non-redundant (NR) database.

ORF[a]	Left	Right	Strand	aa length	Top hit in the NR database	E-value	aa identity (%)
1	126	1145	-	339	tyrosine-type recombinase/integrase [*Phyllobacterium* sp. SYP-B3895]	1.1E-174	68.6
2	1126	1326	-	66	hypothetical protein [*Rhizobium* sp. L51/94]	1.4E-22	65.2
3	1323	1601	-	92	hypothetical protein [*Aquamicrobium aerolatum*]	7.6E-41	70.7
4	1598	1771	-	57	hypothetical protein [Xanthobacter sp. YC-JY1]	4.1E-22	74.1
5	1783	2502	-	239	hypothetical protein [*Ensifer* sp. PDNC004]	8.7E-76	55.7
6	2499	3641	-	380	phage Gp37/Gp68 family protein [*Georhizobium profundi*]	5.2E-157	60.5
7	3634	3873	-	79	hypothetical protein [*Aquamicrobium* sp. NLF2-7]	2.1E-15	63.6
10	4582	4839	+	85	hypothetical protein [*Agrobacterium vitis*]	2.6E-17	51.3
11	4991	5551	-	186	XRE family transcriptional regulator [*Mesorhizobium atlanticum*]	9.3E-95	71.7
12	5649	5864	+	71	XRE family transcriptional regulator [*Rhizobium* sp. Q54]	2.9E-19	61.7
14	6484	6981	+	165	hypothetical protein [*Mesorhizobium* sp. Root157]	6.1E-76	69.1
15	6981	7301	+	106	hypothetical protein [*Pseudaminobacter arsenicus*]	8.2E-26	59.8
16	7301	7618	+	105	hypothetical protein [*Pseudaminobacter arsenicus*]	5.7E-43	73.5
17	7618	7950	+	110	DUF2312 domain-containing protein [*Mesorhizobium* sp. NBSH29]	1.1E-52	78.9
18	7950	8600	+	216	MT-A70 family methyltransferase [*Agrobacterium rhizogenes*]	1.1E-54	62.8
19	8597	9748	+	383	MT-A70 family methyltransferase [*Mesorhizobium alhagi*]	0	78.2
20	9745	10044	+	99	hypothetical protein [*Nitratireductor pacificus*]	3.3E-38	68.9
21	10031	10324	+	97	hypothetical protein [*Aminobacter* sp. SR38]	8.8E-16	45.4
22	10321	10605	+	94	hypothetical protein [*Mesorhizobium xinjiangense*]	3.8E-28	71.8
24	11038	11616	+	192	hypothetical protein [*Aquamicrobium zhengzhouense*]	1.2E-33	42.3
25	11613	13655	+	680	DNA cytosine methyltransferase [*Mesorhizobium* sp. M1A.F.Ca.IN.020.03.2.1]	0	71.0
26	13658	13972	-	104	DUF982 domain-containing protein [*Rhizobiaceae* bacterium]	1.08E-09	46.5
27	14038	15294	+	418	hypothetical protein [*Pararhizobium haloflavum*]	5.8E-122	54.4

28	15263	15952	+	229	hypothetical protein [*Mesorhizobium* sp. NBSH29]	1.3E-85	67.4
29	16081	16416	+	111	HNH endonuclease [*Pseudaminobacter arsenicus*]	3.2E-46	74.3
30	16530	17012	+	160	helix-turn-helix domain-containing protein [*Rhodobiaceae* bacterium]	5.5E-83	76.3
31	16999	18711	+	570	terminase large subunit [*Mesorhizobium* sp.]	0	88.3
32	18708	19943	+	411	phage portal protein [*Devosia geojensis*]	0	74.7
33	19954	20643	+	229	HK97 family phage prohead protease [*Mesorhizobium* sp. Root552]	8.5E-128	84.2
34	20663	22009	+	448	phage major capsid protein [*Devosia geojensis*]	0	82.9
35	22082	22294	+	70	hypothetical protein [*Devosia geojensis*]	1.2E-22	66.7
36	22297	22857	+	186	head-tail connector protein [*Ensifer adhaerens*]	2.0E-56	55.2
37	22857	23210	+	117	head-tail adaptor protein [*Mesorhizobium* sp. B1-1-7]	1.4E-49	71.7
38	23367	23810	+	147	HK97 gp10 family phage protein [*Sphingomonas* sp.]	1.9E-44	55.0
39	23807	24193	+	128	DUF3168 domain-containing protein [*Rhizobium* sp. KVB221]	7.1E-45	58.4
40	24212	24667	+	151	phage tail tube protein [*Rhizobium* sp. KVB221]	6.0E-69	68.7
41	24664	25089	+	141	gene transfer agent family protein [*Sphingomonas* sp.]	3.6E-53	66.9
42	25089	25238	+	49	hypothetical protein [*Rhizobium* sp. CG5]	9.8E-12	64.6
43	25235	25528	-	97	hypothetical protein [*Neorhizobium* sp. NCHU2750]	2.6E-18	58.3
44	25543	27717	+	724	tape measure protein [*Nitratireductor kimnyeongensis*]	4.9E-179	52.6
45	27720	28367	+	215	hypothetical protein [*Phyllobacterium leguminum*]	6.8E-59	52.3
46	28364	29050	+	228	hypothetical protein [*Ochrobactrum* sp. GRS2]	6.9E-79	59.0
47	29047	29484	+	145	hypothetical protein [*Ochrobactrum* sp. Marseille-Q0166]	2.2E-61	70.3
49	29707	30021	+	104	phage tail protein [*Rhizobium* sp. AB2/73]	8.7E-04	59.5
52	30603	34070	+	1155	phage tail protein [*Rhizobium* sp. AB2/73]	0	46.8
54	34315	34668	+	117	hypothetical protein [*Nitratireductor indicus*]	3.0E-21	66.3
55	34665	35915	+	416	hypothetical protein [*Marinobacter* sp. 3-2]	2.5E-35	38.7
56	35937	36563	-	208	class I SAM-dependent methyltransferase [*Chelativorans oligotrophicus*]	4.6E-58	47.6
57	36784	37545	+	253	N-acetylmuramoyl-L-alanine amidase [*Rhizobium subbaraonis*]	1.4E-118	67.6

59	37945	38118	+	57	hypothetical protein [*Rhizobium* sp. 9140]	8.4E-11	69.6
60	38184	38786	+	200	hypothetical protein [*Sinorhizobium fredii*]	3.4E-88	66.5
61	38809	39249	-	146	hypothetical protein [*Mesorhizobium* sp. M3A.F.Ca.ET.175.01.1.1]	2.9E-10	50
62	39629	40561	+	310	hypothetical protein [*Agrobacterium tumefaciens*]	1.6E-174	77.3

[a]Virion proteins detected by mass spectrometry were indicated in red.

Table S2. Predicted ORFs in the vB_MseS-P1 genome and four reference phage genomes and their putative functions determined on the basis of conserved domains and remote homologs.

ORF[a]	Strand	Left	Right	aa length	Putative function[b]	Conserved domain (E-value)[c]	HHpred search[d] Significant Hits[e]	Probability (%)	E-value
**vB_MseS-P1**									
1	-	126	1145	339	tyrosine-type integrase	cd00800 (6.6E-26)			
2	-	1126	1326	66					
3	-	1323	1601	92	ferric uptake regulation protein		Ferric uptake regulation protein [*Francisella tularensis*]	93	0.3
4	-	1598	1771	57					
5	-	1783	2502	239	ASCH domain containing protein		ASCH domain containing protein [*Escherichia coli*]	98.7	1.2E-06
6	-	2499	3641	380	spore photoproduct lyase		Spore photoproduct lyase [*Geobacillus thermoden*]	98.2	2.0E-05
7	-	3634	3873	79			Talin [*Gallus gallus*]	91.9	1.1
8	-	3878	4138	86					
9	-	4140	4400	86					
10	+	4582	4839	85					
11	-	4991	5551	186	LexA repressor	cd00093 (1.6E-13)			
12	+	5649	5864	71	repressor protein C	cd00093 (2.7E-04)			
13	+	5861	6361	166	transcriptional repressor		Omega transcriptional repressor [*Streptococcus pyogenes*]	90.5	1.3
14	+	6484	6981	165	CII repressor		Regulatory protein CII [*Escherichia* phage 186]	99.9	3.1E-21
							Repressor protein C [*Rhizobium* phage 16-3]	96.9	9.1E-03
							lambda C1 repressor [Bacteriophage lambda]	96.7	3.5E-02
15	+	6981	7301	106	GcrA cell cycle regulator		GcrA cell cycle regulator [*Caulobacter vibrioides* NA1000]	99.5	4.5E-14

16	+	7301	7618	105	phage repressor		Accessory factor PcfF [*Enterococcus faecalis*]	99.5	7.6E-13
							Arc/Mnt-like phage repressor [*Salmonella* bacteriophage P22]	98.6	5.6E-07
17	+	7618	7950	110	DNA-binding protein		DNA-binding protein involved in cell-division [*Caulobacter vibrioides* ATCC 19]	99.9	6.8E-23
18	+	7950	8600	216	chromosome segregation protein ParB	cd16404 (1.7E-13)			
19	+	8597	9748	383	N6-adenosine-specific RNA methylase IME4	COG4725 (7.4E-28)			
20	+	9745	10044	99					
21	+	10031	10324	97	phage repressor		Antitoxin HigA [*Escherichia coli*]	98.7	1.4E-06
							Transcriptional regulator ComR [*Streptococcus vestibularis* F0396]	98.4	1.7E-05
							Repressor protein C [*Rhizobium* phage 16-3]	98.4	1.7E-05
22	+	10321	10605	94					
23	+	10605	11045	146	chromosomal replication initiation factor DnaA		Chromosomal replication initiation factor DnaA C-terminal domain IV [*Aquifex aeolicus*]	99.5	3.1E-13
24	+	11038	11616	192					
25	+	11613	13655	680	site-specific DNA-cytosine methylase	COG0270 (5.9E-34)			
26	-	13658	13972	104	DUF982 domain-containing protein	pfam06169 (6.5E-06)			
27	+	14038	15294	418	DNA replication initiation protein		Replication protein repL [*Escherichia* phage P1]	98.3	2.2E-05
							Chromosome replication initiation protein [*Geobacillus kaustophilus* HTA426]	98.0	1.6E-04

28	+	15263	15952	229	transcription termination/antitermination factor NusG	COG0250 (4.7E-14)			
29	+	16081	16416	111	HNH nucleases	cd00085 (1.2E-05)			
**30**	+	16530	17012	160	terminase small subunit		terminase small subunit [*Shigella* phage Sf6]	98.7	8.7E-07
**31**	+	16999	18711	570	terminase large subunit	COG4626 (2.2E-100)	Putative terminase large subunit [*Shigella* phage SfV]	100	3.9E-49
							large terminase [*Escherichia* virus HK97]	100	1.2E-41
							terminase large subunit [*Escherichia* phage lambda]	99.9	1.3E-18
**32**	+	18708	19943	411	portal protein	pfam04860 (1.3E-113), TIGR01537 (3.9E-81)	Portal protein [Enterobacteria phage HK97]	100	1.3E-41
							Phage portal protein Rcc01684, HK97 family, g3 [*Rhodobacter capsulatus*]	100	3.8E-39
**33**	+	19954	20643	229	prohead protease	TIGR01543 (3.6E-40)	Prohead protease [Enterobacteria phage HK97]	100.0	2.3E-29
**34**	+	20663	22009	448	major capsid protein	TIGR01554 (8.9E-56)	Major capsid protein [Enterobacteria phage HK97]	100	2.4E-40
							Major capsid protein Rcc01687, g5 [*Rhodobacter capsulatus* DE442]	100	8.9E-32
**35**	+	22082	22294	70	DNA-packaging protein FI		DNA-packaging protein FI [*Escherichia* phage lambda]	96.4	2.0E-02
**36**	+	22297	22857	186	adaptor	cd08054 (4.5E-13)	Adaptor protein Rcc01688, g6 [*Rhodobacter capsulatus*]	100.0	2.9E-25
							Head completion protein [*Escherichia* phage T5]	99.9	6.9E-21
							head-tail connector [Enterobacteria phage HK97]	99.2	1.6E-10

**37**	+	22857	23210	117	stopper	pfam05521 (4.3E-07), TIGR01563 (6.0E-05)	Stopper protein Rcc01689, g7 [*Rhodobacter capsulatus*]	99.9	1.2E-19
							Head completion protein gp16 [*Bacillus* phage SPP1]	99.8	9.6E-20
							Head-tail connector protein FII [*Escherichia* phage lambda]	96.0	0.2
**38**	+	23367	23810	147	tail completion protein		Putative capsid assembly protein G [*Escherichia* phage Mu]	98.9	1.0E-08
							Tail completion protein S [*Escherichia* phage P2]	97.9	1.0E-04
							Tail completion protein Z [*Escherichia* phage lambda]	95.3	0.3
**39**	+	23807	24193	128	tail terminator		Tail terminator protein Rcc01690, g8 [*Rhodobacter capsulatus*]	99.9	3.2E-20
							Tail completion protein gp17 [*Bacillus* phage SPP1]	99.8	3.8E-18
							Tail tube terminator protein [*Escherichia* phage lambda]	98.6	6.2E-06
**40**	+	24212	24667	151	tail tube protein	pfam06199 (5.0E-14)	Phage major tail protein, TP901-1 family, Rcc01691, g9 [*Rhodobacter capsulatus*]	99.8	7.2E-18
							Major tail protein V [Enterobacteria phage lambda]	99.7	5.4E-16
**41**	+	24664	25089	141	TAC_11 family tail assembly protein, similar to RcGTA-gp10	pfam11836 (1.4E-13)	conserved hypothetical protein [*Pseudomonas aeruginosa* PAO1]	90.4	3.3
**42**	+	25089	25238	49					
**43**	-	25235	25528	97					

**44**	+	25543	27717	724	tail tape measure protein	TIGR02675 (5.2E-13), TIGR01541 (3.3E-09)	Tape measure protein [*Escherichia* phage lambda]	99.1	3.8E-05
**45**	+	27720	28367	215	distal tail		Distal tail protein [*Escherichia* virus T5]	100	6.9E-33
							Baseplate of native GTA particle, Rcc01695, g12 [*Rhodobacter capsulatus*]	98.0	1.6E-03
**46**	+	28364	29050	228	hub		Baseplate hub protein pb3 [*Escherichia* phage T5]	99.9	4.7E-21
							Baseplate of native GTA particle, Rcc01696, g13 [*Rhodobacter capsulatus*]	99.7	1.1E-16
**47**	+	29047	29484	145	peptidase		NlpC/P60 family cysteine proteinases [*Anabaena variabilis*]	98.4	2.1E-05
							Tail tip assembly protein K, peptidase C40 family [*Escherichia* phage lambda]	97.8	5.0E-04
**48**	+	29477	29665	62					
**49**	+	29707	30021	104					
**50**	+	30024	30260	78					
**51**	-	30261	30527	88					
**52**	+	30603	34070	1155	megatron	pfam13550 (2.5E-09)	Probable baseplate hub protein [*Escherichia* phage T5]	100	4.0E-47
							Tip attachment protein J [*Escherichia* phage lambda]	100	7.8E-43
							Baseplate of native GTA particle, Rcc01698, g15 [*Rhodobacter capsulatus*]	100.0	1.6E-27
53	+	34078	34311	77	proteasome-associated ATPase		Proteasome-associated ATPase [*Mycobacterium tuberculosis* H37Rv]	96.2	0.1
54	+	34315	34668	117	tail fiber protein		Probable tail fiber protein [*Escherichia* phage T1]	97.3	8.6E-03

							DpK2 bacteriophage tail spike depolymerase [*Klebsiella* phage GH-K3]	95.4	0.5
55	+	34665	35915	416	tail fiber protein	pfam03629 (5.6E-13)	Acetylxylan esterase related enzyme [*Clostridium acetobutylicum*]	100.0	1.4E-30
							Non-contractile tail sheath [Enterobacteria phage N4]	99.2	6.1E-11
							Tail fiber protein [*Escherichia* phage vB_EcoP_G7C]	96.3	0.4
56	-	35937	36563	208	S-adenosylmethionine-dependent methyltransferases	pfam08241 (2.7E-14)			
57	+	36784	37545	253	lysozyme	cd06583 (1.0E-24), PHA00447 (6.9E-51)	lysozyme (Zn amidase) [Enterobacteria phage T7]	99.8	5.5E-19
58	+	37591	37908	105	lytic conversion lipoprotein		Lytic conversion lipoprotein [*Escherichia* phage T5]	90.1	0.6
59	+	37945	38118	57					
60	+	38184	38786	200	lysozyme		Putative lysozyme [*Bacillus* phage SF6]	94.9	5.4
							Holin-like protein 24.1 [*Bacillus* phage SPP1]	94.1	1.7
61	-	38809	39249	146					
62	+	39629	40561	310			gene 40 [Enterobacteria phage phi80]	98.8	3.5E-08
63	+	40570	40689	39					
***Rhizobium* phage 16-3**									
**1**	+	46	510	154	terminase small subunit		Terminase small subunit [Enterobacteria phage HK97]	100.0	5.8E-28
**2**	+	523	2100	525	terminase large subunit	COG4626 (4.2E-75)	Large terminase [*Escherichia* virus HK97]	100	1.7E-49
**3**	+	2202	3392	396	portal protein	pfam04860 (4.8E-97), TIGR01537 (1.1E-57)	Portal protein [Enterobacteria phage HK97]	100	5.4E-41

							Phage portal protein Rcc01684, HK97 family, g3 [*Rhodobacter capsulatus*]	100	3.4E-39
**4**	+	3389	4033	214	prohead protease	pfam04586 (7.6E-63), TIGR01543 (2.1E-42)	Prohead protease [Enterobacteria phage HK97]	99.9	4.2E-23
**5**	+	4109	5375	422	major capsid protein	TIGR01554 (4.6E-84), pfam05065 (6.9E-49)	Major capsid protein [Enterobacteria phage HK97]	100	9.3E-37
							Major capsid protein Rcc01687, g5 [*Rhodobacter capsulatus* DE442]	100	8.7E-33
**7**	-	5064	5393	109					
**8**	+	5423	5653	76	DNA-packaging protein FI		DNA-packaging protein FI [Enterobacteria phage lambda]	95.1	0.1
**9**	+	5613	6158	181	adaptor	TIGR02215 (9.1E-10)	Adaptor protein Rcc01688, g6 [*Rhodobacter capsulatus*]	100.0	1.0E-29
							head-tail connector [Enterobacteria phage HK97]	99.3	1.0E-10
**10**	+	6177	6602	141	capsid fiber protein		Capsid fiber protein [*Bacillus* phage phi29]	98.3	5.0E-05
							Capsid decoration protein [*Escherichia* phage Lambda]	90.2	4.2
**11**	+	6604	6951	115	stopper	pfam05521 (2.1E-09)	Stopper protein Rcc01689, g7 [*Rhodobacter capsulatus*]	99.9	5.3E-20
							Head-tail connector protein FII [*Escherichia* phage lambda]	96.0	0.2
**12**	+	6951	7367	138	tail completion protein, HK97 gp10 family	pfam04883 (2.9E-08), TIGR01725 (2.1E-06)	capsid assembly protein G [*Escherichia* phage Mu]	99.0	2.1E-09
							Tail completion protein S [*Escherichia* phage P2]	97.9	7.9E-05

						Tail completion protein Z [*Escherichia* phage lambda]	95.3	0.4
**13**	+	7386	7793	135	tail terminator	pfam11367 (1.8E-28)	Tail terminator protein Rcc01690, g8 [*Rhodobacter capsulatus*]	99.9	2.8E-21
							Tail tube terminator protein [*Escherichia* phage lambda]	98.2	3.6E-04
**14**	+	7793	7978	61					
**15**	+	8028	8474	148	tail tube protein	pfam06199 (3.5E-20)	Phage major tail protein, TP901-1 family, Rcc01691, g9 [*Rhodobacter capsulatus*]	99.8	2.0E-18
							Major tail protein V [Enterobacteria phage lambda]	99.7	4.2E-16
**16**	+	8478	8831	117	TAC_11 family tail assembly protein, GTA-gp10	pfam11836 (3.0E-23)	Uncharacterized 11.3 kDa protein in lys 3'region [*Haemophilus* phage HP1]	92.8	2.9
**17**	+	8888	9040	50	tail assembly protein		Tail assembly protein E' [*Escherichia* phage P2]	94.8	0.1
**18**	+	9267	11846	859	tail tape measure protein	pfam06791 (2.2E-09)	Tape measure protein [*Escherichia* phage N15]	99.6	2.1E-08
							Tape measure protein [*Escherichia* phage lambda]	99.5	2.5E-08
**19**	+	11837	12541	234	distal		Distal tail protein [*Escherichia* phage T5]	100.0	2.1E-26
							Baseplate of native GTA particle, Rcc01695, g12 [*Rhodobacter capsulatus*]	96.9	0.2
**20**	+	12551	12709	52			Uncharacterized 8.9 kDa protein in int-C1 intergenic region [*Haemophilus* phage HP1]	99.5	7.9E-14
**21**	+	12713	13285	190	hub		Probable baseplate hub protein [*Escherichia* phage T5]	99.8	6.3E-20
							Baseplate of native GTA particle, Rcc01696, g13 [*Rhodobacter capsulatus*]	99.6	2.1E-14
							baseplate hub protein [*Escherichia* phage lambda]	99.2	2.4E-10

**22**	+	13270	13680	136	peptidase		Tail tip assembly protein K [*Escherichia* phage N15]	98.2	1.7E-05
							Tail tip assembly protein K [*Escherichia* phage lambda]	97.2	7.8E-03
**23**	+	13703	15814	703	megatron		TIPJ_LAMBD Tip attachment protein J [*Escherichia* phage lambda]	100	1.0E-40
							Baseplate of native GTA particle, Rcc01698, g15 [*Rhodobacter capsulatus*]	100.0	7.0E-25
24	+	15889	18300	803	tail spike protein		Tail spike protein [Acinetobacter phage vB_AbaP_AS12]	96.9	5.7E-02
25	-	18306	18689	127	acetyltransferase	pfam13302 (2.0E-13)			
26	+	18390	18740	116					
27	-	18737	18916	59					
28	+	19039	19392	117	S-adenosyl-L-methionine-dependent methyltransferase		S-adenosyl-L-methionine-dependent tRNA methyltransferase [*Yarrowia lipolytica* CLIB122]	99.2	8.8E-10
29	+	19471	19962	163	lysozyme		Lysozyme-like Family 19 glycosidase [*Carica papaya*]	95.8	0.3
30	+	19925	20071	48					
31	+	20099	20260	53					
32	+	20319	20582	87					
33	+	20563	21066	167	spanin, inner membrane subunit		Spanin, inner membrane subunit [Enterobacteria phage T4]	97.2	8.2E-02
34	+	20579	20731	50					
35	+	21218	21484	88	rubredoxin		Rubredoxin [*Mycobacterium ulcerans*]	93.9	3.7E-02
36	+	21624	22523	299	trypsin-like (serine) peptidase	pfam13365 (4.5E-19)			
37	+	22520	22654	44					

38	+	22651	23067	138	anti-phage defense ZorAB system ZorA	NF033917 (4.0E-04)			
39	+	23085	23375	96					
40	+	23369	23905	178	serine protease		Alkaline serine protease kp-43, C-terminal domain [*Bacillus* sp. KSM-KP43]	98.1	2.9E-04
41	+	23905	24090	61					
42	+	24087	24512	141					
43	+	24625	24990	121					
44	+	24987	26249	420	DNA polymerase II small subunit		DNA polymerase II small subunit [*Pyrococcus abyssi*]	99.5	8.0E-13
							Probable exonuclease subunit 1 [*Escherichia* phage T5]	99.4	3.4E-12
45	+	26246	26485	79					
46	+	26538	26756	72	DUF2829 domain containing protein	pfam11195 (9.7E-11)			
47	+	26753	27478	241	phosphoprotein phosphatase	cd00144 (2.5E-20)			
48	+	27475	28242	255	5'-deoxynucleotidase YfbR	COG1896 (4.0E-07)			
49	+	28260	28460	66					
50	+	28457	28819	120					
51	+	28816	29484	222	deoxynucleoside monophosphate kinase		Deoxynucleoside monophosphate kinase [Bacteriophage T4]	99.8	2.0E-18
52	+	29472	29672	66					
53	-	29739	30044	101	R67 dihydrofolate reductase		R67 dihydrofolate reductase [*Escherichia coli*]	94.6	0.3
54	-	30783	31898	371	integrase	cd00796 (2.7E-08)			
55	-	31673	32095	140	excisionase		Excisionase [*Shigella* phage SfV]	99.1	1.2E-09
56	-	32086	32448	120	NHN endonuclease	PHA00280 (9.0E-09)			

57	-	32385	32654	89	homing endonuclease		endonuclease [Enterobacteria phage T3]	99.2	1.5E-10
58	-	32651	32899	82					
59	-	32896	33243	115			Uncharacterized 8.6 kDa protein in ral-gp17 intergenic region [*Salmonella* phage P22]	99.7	2.7E-15
60	-	33554	33697	47					
61	-	33697	33819	40					
62	+	33782	34165	127	transcription elongation factor SPT5		Hyper conserved RNA bingding Protein [*Prochlorococcus marinus* str. MIT 9303]	97.0	5.1E-03
							Transcription elongation factor SPT5 [*Methanocaldococcus jannaschii* DSM 2661]	93.6	0.7
63	-	34168	34425	85			Uncharacterized 17.8 kDa protein in arn-motA intergenic region [Enterobacteria phage T4]	100.0	3.4E-30
64	+	34346	34480	44					
65	-	34422	34817	131					
66	-	34877	35347	156	restriction alleviation protein Lar	TIGR03655 (4.6E-09)			
67	-	35673	36464	263	LexA repressor	COG2932 (4.0E-21)	Repressor protein C [*Rhizobium* phage 16-3]	100.0	1.5E-25
68	+	35703	35936	77					
69	+	36556	36723	55					
70	-	37067	37678	203	LexA repressor		Repressor protein C [*Rhizobium* phage 16-3]	99.9	7.6E-23
71	+	37115	37693	192					
72	+	37858	38154	98	middle operon regulator, Mor		Middle operon regulator, Mor [Bacteriophage Mu]	95.8	6.3E-02
							Regulatory protein CII [Bacteriophage lambda]	93.7	0.7
73	+	38151	38468	105					
74	+	38468	39295	275	transcription elongation factor SPT5		Transcription elongation factor SPT5 [*Saccharomyces cerevisiae* S288C]	95.2	0.6

75	+	39366	39626	86					
76	+	39619	39861	80	membrane protein		Putative membrane protein [*Geobacillus thermodenitrificans* NG80-2]	92.6	0.5
77	+	40023	40814	263	CRISPR/Cas system-associated protein Cas3	pfam13479 (6.9E-41)		99.4	1.3E-10
								99.3	2.2E-10
78	+	41062	41685	207					
79	+	41754	42155	133	lytic conversion lipoprotein		Lytic conversion lipoprotein [*Escherichia* phage T5]	94.1	6.7E-02
80	+	42167	42568	133			Heterocyst differentiation control protein [*Nostoc* sp. PCC 7120]	94.5	0.1
81	+	42583	43719	378	DNA polymerase III subunit beta	PRK05643 (3.0E-137)			
82	+	43719	43994	91					
83	+	44015	44332	105					
84	+	44342	45016	224	HNH endonuclease	pfam14279 (1.0E-10)	Restriction endonuclease [*Pseudomonas alcaligenes*]	99.2	4.3E-11
							Endonuclease VII [*Mycobacterium* phage D29]	99.1	8.6E-10
85	+	45017	45970	317	exonuclease	cd22343 (1.9E-04)	Exonuclease [*Escherichia* phage lambda]	99.4	7.4E-12
86	+	45996	46496	166	RNA polymerase sigma factor 70	TIGR02937 (7.4E-09)			
87	+	46493	47008	171	RNA polymerase sigma factor 70	TIGR02937 (1.9E-09)			
88	+	47008	48738	576	superfamily II DNA or RNA helicase	COG1061 (1.5E-46)			
89	+	49017	49244	75					

90	+	49241	49522	93					
91	+	49513	50271	252	DNA ends protecting protein gam		Putative DNA ends protecting protein gam [*Escherichia* phage Mu]	91.9	3.9
92	+	50271	50576	101					
93	+	50576	50737	53					
94	+	50734	51090	118	transcriptional repressor, PadR-like family		Transcriptional repressor PadR-like family PadR [*Bacillus cereus* ATCC 14579]	97.1	2.3E-03
95	+	51091	51285	64					
96	+	51381	52022	213	DNA modification methylase		S-adenosyl-L-methionine-dependent methyltransferase [*Bacillus cereus* ATCC 10987]	99.2	2.0E-10
							DNA N-6-adenine-methyltransferase [*Haemophilus* phage HP1]	99.1	5.7E-09
97	+	52013	52684	223	DNA modification methylase	COG0863 (1.1E-23)			
98	+	52681	53055	124	DNA gyrase inhibitor YacG		DNA gyrase inhibitor YacG [*Escherichia coli*]	90.6	0.7
99	+	53120	53329	69	CII repressor	TIGR03830 (1.9E-06)	Repressor protein C [*Rhizobium* phage 16-3]	96.8	3.7E-02
100	+	53283	54080	265	HNH nucleases	cd00085 (7.3E-03)	Restriction endonuclease PacI [*Pseudomonas alcaligenes*]	98.8	6.2E-09
							Recombination endonuclease VII, N-terminal domain [Bacteriophage T4]	97.7	1.2E-04
101	+	54081	56249	722	bifunctional DNA primase/polymerase	pfam09250 (4.4E-34)			
102	-	56607	57425	272					
103	+	56685	57194	169	VRR-NUC	smart00990 (4.8E-05)			
104	+	57225	57548	107					

105	+	57548	57691	47			Zinc finger domain-containing protein [*Chaetomium thermophilum* var. *thermophilum* DSM 1495]	91.3	0.8
106	+	57830	57985	51					
107	+	57975	58667	230					
108	+	58664	59413	249	HNH endonuclease	cd00085 (9.5E-08)			
109	+	59553	59855	100					
110	+	59848	60078	76	HNH endonuclease	pfam01844 (1.4E-04)			
***Escherichia* phage HK97**									
**1**	+	50	535	161	terminase small subunit		Terminase small subunit [Enterobacteria phage HK97]	100	9.7E-36
**2**	+	542	2056	504	terminase large subunit	COG4626 (7.4E-82)	Terminase large subunit [*Escherichia* virus HK97]	100	2.1E-51
**3**	+	2056	3330	424	portal	pfam04860 (1.4E-110), TIGR01537 (5.1E-77)	Portal protein [Enterobacteria phage HK97]	100	1.2E-46
							Phage portal protein Rcc01684, HK97 family, g3 [*Rhodobacter capsulatus*]	100	1.8E-41
**4**	+	3348	4025	225	prohead protease	TIGR01543 (3.6E-67)	Prohead protease [Enterobacteria phage HK97]	100	1.1E-35
**5**	+	4028	5185	385	major capsid	TIGR01554 (6.1E-61), pfam05065 (1.2E-48)	Major capsid protein Rcc01687, g5 [*Rhodobacter capsulatus* DE442]	100	1.2E-31
**6**	+	5219	5545	108	adaptor	cd08051 (1.9E-33)	Adaptor protein Rcc01688, g6 [*Rhodobacter capsulatus*]	99.2	9.0E-10
**7**	+	5545	5883	112	stopper	COG5614 (5.4E-32), pfam05521 (3.2E-19)	Stopper protein Rcc01689, g7 [*Rhodobacter capsulatus*]	99.9	4.7E-20

**8**	+	5880	6329	149	tail completion protein	TIGR01725 (5.8E-24), pfam04883 (4.5E-07)	Putative capsid assembly protein G [*Escherichia* phage Mu]	99.3	1.2E-10
							Tail completion protein S [*Escherichia* phage P2]	98.2	4.2E-05
**9**	+	6326	6673	115	tail terminator	pfam11367 (2.9E-07)	Tail terminator protein Rcc01690, g8 [*Rhodobacter capsulatus*]	99.6	4.3E-14
**10**	+	6733	7437	234	tail tube	pfam00047 (1.4E-03)	Tail tube protein [*Escherichia* phage lambda]	99.4	6.5E-10
							Phage major tail protein, TP901-1 family, Rcc01691, g9 [*Rhodobacter capsulatus*]	99.4	9.8E-11
**11**	+	7472	8145	224	TAC 1 tail assembly protein	pfam06222 (6.2E-70)	Tail assembly chaperone [*Escherichia* virus HK97]	99.9	2.2E-23
**13**	+	8203	8391	62	spanin, inner membrane subunit	pfam13974 (8.6E-06)	Probable spanin, inner membrane subunit [*Escherichia* phage P2]	95.4	6.8E-01
**14**	+	8435	11704	1089	tail tape measure protein	COG5281 (1.8E-70)	Tape measure protein [*Escherichia* phage T1]	99.9	7.2E-13
							Tape measure protein [*Escherichia* phage lambda]	99.7	1.1E-10
**15**	+	11707	12045	112	distal tail	pfam05939 (8.6E-48), COG4718 (2.2E-33)	Tail tip protein M [*Escherichia* phage lambda]	100.0	2.2E-27
							Baseplate of native GTA particle, Rcc01695, g12 [*Rhodobacter capsulatus*]	99.6	3.0E-15
**16**	+	12042	12800	252	hub	pfam05100 (1.5E-102), COG4672 (1.8E-94)	Tail tip protein L [*Escherichia* phage N15]	100	6.8E-40
							Baseplate of native GTA particle, Rcc01696, g13 [*Rhodobacter capsulatus*]	99.9	4.0E-22
**17**	+	12802	13512	236	peptidase	cd08073 (5.0E-56), pfam00877 (2.1E-27)	Tail tip assembly protein K [*Escherichia* phage N15]	100	3.9E-35

**18**	+	13834	14442	202	tail tip assembly protein I	COG4723 (2.0E-62)	Tail tip assembly protein I [*Escherichia* phage N15]	100	1.2E-34
**19**	+	14475	14837	120			no hit		
**20**	-	14715	14975	86			no hit		
**21**	+	15141	19031	1296	megatron	COG4733 (0), pfam13550 (5.8E-13)	Tip attachment protein J [*Escherichia* phage lambda]	100	3.4E-108
							Baseplate of native GTA particle, Rcc01698, g15 [*Rhodobacter capsulatus*]	99.6	1.9E-13
22	+	19239	20204	321	tail fiber protein	pfam07484 (6.0E-15)	Receptor-binding domain of short tail fibre protein gp12 [Bacteriophage T4]	96.6	0.1
23	+	20204	20812	202	tail fibre assembly protein	pfam02413 (3.7E-37)	Tail fiber assembly protein [*Escherichia* phage lambda]	100.0	2.6E-28
24	-	21318	22388	356	integrase	cd00800 (1.4E-63)	Integrase [Enterobacteria phage lambda]	100	1.6E-44
25	-	22366	22584	72	excisionase	pfam07825 (1.3E-37)	Excisionase [*Escherichia* phage HK022]	99.6	2.0E-13
26	-	22772	23302	176	Eae protein		Eae protein [*Salmonella* phage P22]	99.2	1.4E-09
27	-	23299	23463	54		pfam10930 (4.6E-22)	Uncharacterized 6.6 kDa protein in eae-abc2 intergenic region [*Salmonella* phage P22]	100	2.5E-33
28	-	23474	23767	97	anti-RecBCD protein 2	pfam11043 (5.5E-61)	Anti-RecBCD protein 2 [*Salmonella* phage P22]	100	1.1E-47
29	-	23786	24175	129	SSDNA-binding protein		single-stranded DNA-binding protein [*Mycobacterium tuberculosis* H37Rv]	99.8	5.2E-18
30	-	24175	24780	201	DNA single-strand annealing protein (SSAP)	pfam04404 (4.8E-43)	Essential recombination function protein [*Salmonella* phage P22]	100	6.0E-34
31	-	24791	25042	83	accessory recombination protein		Accessory recombination function protein [*Salmonella* phage P22]	90.3	0.2
32	-	24791	24967	58			no hit		
33	-	25039	25191	50	protein kil		Protein kil [*Escherichia* phage HK022]	100.0	1.4E-29

34	-	25176	25310	44	regulatory protein CIII	pfam08134 (8.8E-09)	Regulatory protein CIII [*Escherichia* phage HK022]	100.0	1.1E-27
35	-	25505	25975	156			no hit		
36	-	26034	26417	127	antitermination protein N		Probable regulatory protein N [*Salmonella* phage P22]	99.6	3.5E-15
							Antitermination protein N [*Escherichia* phage lambda]	98.7	1.5E-09
37	-	26909	27559	216	Holin-like protein		Holin-like protein 24.1 [*Bacillus* phage SPP1]	97.5	7.5E-03
38	-	27547	27813	88			no hit		
39	-	28239	28952	237	LexA repressor	COG1974 (3.6E-57)	Repressor protein cI [*Escherichia* phage lambda]	100.0	1.1E-27
						pfam00717 (7.1E-35)	Repressor protein C [*Rhizobium* phage 16-3]	99.9	1.1E-22
40	+	29053	29253	66	antirepressor Cro	pfam09048 (4.8E-33)			
41	+	29391	29687	98	CII repressor	pfam05269 (9.3E-30)			
42	+	29720	29881	53			no hit		
43	+	29868	30689	273	replication protein O	pfam04492 (1.8E-40)			
44	+	30686	32062	458	Replicative DNA helicase	cd00984 (2.1E-121)			
45	+	32059	32157	32			no hit		
46	+	32144	32356	70			no hit		
47	+	32337	32777	146	NinB protein	pfam05772 (1.9E-59)			
48	+	32774	33301	175	DNA N-6-adenine-methyltransferase	TIGR01712 (3.5E-88)			
49	+	33298	33480	60	NinE protein	pfam05322 (2.2E-23)			
50	+	33477	33647	56	NinF protein	pfam05810 (2.0E-22)			
51	+	33640	34365	241	antirepressor protein	COG3645 (9.5E-53)			
						COG3646 (1.4E-23)			
52	+	34365	34655	96	nuclease ybcO	pfam07102 (1.7E-50)	Putative nuclease ybcO [Enterobacteria phage 82]	100.0	2.0E-26

53	+	34652	35014	120	Endodeoxyribonuclease RusA	PRK09786 (9.5E-43)			
54	+	35011	35199	62	NinH protein	pfam06322 (1.7E-24)			
55	+	35196	35819	207	Antitermination protein	pfam03589 (8.3E-33)			
56	+	36248	36568	106	holin	TIGR01594 (4.1E-37)			
57	+	36552	37028	158	lysin	COG4678 (1.7E-84)			
58	+	37025	37462	145	Rz lysis protein	pfam03245 (1.9E-34)			
59	+	37672	38400	242	KilA-N domain containing protein	pfam04383 (2.0E-25)			
60	+	39054	39413	119	HNH nucleases	cd00085 (6.5E-06)			
***Escherichia* phage Lambda**									
**1**	+	191	736	181	terminase small subunit	pfam07471 (1.1E-92)	Terminase small subunit [*Escherichia* phage lambda]	99.9	2.1E-22
**2**	+	711	2636	641	terminase, large subunit	COG5525 (0)	Terminase, large subunit [*Escherichia* phage lambda]	100	1.2E-88
**3**	+	2633	2839	68	head completion protein	pfam02831 (2.5E-24)	Head completion protein [*Escherichia* phage lambda]	99.8	2.6E-19
**4**	+	2836	4437	533	portal protein	COG5511 (0); TIGR01539 (1.6E-150)	Phage portal protein Rcc01684, HK97 family, g3 [*Rhodobacter capsulatus*]	99.9	3.2E-19
**5**	+	4418	5737	439	capsid assembly protease C	cd07022 (9.7E-90)	Capsid assembly protease C [*Escherichia* phage lambda]	100	4.2E-51
**6**	+	5132	5737	201	capsid assembly protein	pfam01343 (6.9E-14)	Capsid assembly protease C [*Escherichia* phage lambda]	99.6	1.2E-13
**7**	+	5747	6079	110	capsid decoration protein	pfam02924 (1.3E-14)	Capsid decoration protein [*Escherichia* phage lambda]	100.0	3.8E-26
**8**	+	6135	7160	341	major capsid protein	pfam03864 (7.9E-78)	Major capsid protein [*Escherichia* phage lambda]	100	2.8E-47

**9**	+	7202	7600	132	DNA packaging protein FI	pfam14000 (5.7E-54)	DNA-packaging protein FI [*Escherichia* phage lambda]	100	2.9E-39
**10**	+	7612	7965	117	stopper	pfam05354 (1.0E-75)	Stopper protein Rcc01689, g7 [*Rhodobacter capsulatus*]	95.4	0.5
**11**	+	7977	8555	192	tail completion protein Z	pfam06763 (2.1E-72)	Tail completion protein Z [*Escherichia* phage lambda]	100.0	5.8E-30
**12**	+	8552	8947	131	tail terminator	pfam06141 (2.8E-71)	Tail terminator protein Rcc01690, g8 [*Rhodobacter capsulatus*]	98.0	1.6E-03
**13**	+	8955	9695	246	tail tube	pfam16461 (1.6E-72)	Phage major tail protein, TP901-1 family, Rcc01691, g9 [*Rhodobacter capsulatus*]	99.7	1.8E-14
**14**	+	9711	10133	140	tail assembly chaperone G, TAC 2	pfam06894 (1.7E-72)	Tail assembly protein G [*Escherichia* phage lambda]	100	1.5E-44
**15**	+	10115	10549	144	tail assembly protein T	pfam06223 (3.2E-58)	Tail assembly protein GT [*Escherichia* phage lambda]	100	3.4E-40
**16**	+	10542	13103	853	tail tape measure protein	COG5281 (0), TIGR01541 (3.8E-104)	Tape measure protein [*Escherichia* phage lambda]	100	1.0E-49
**17**	+	13100	13429	109	distal tail	COG4718 (8.7E-58), pfam05939 (3.5E-49)	Baseplate of native GTA particle, Rcc01695, g12 [*Rhodobacter capsulatus*]	99.7	2.5E-16
							Distal tail protein [*Escherichia* phage T5]	96.6	4.0E-02
**18**	+	13429	14127	232	hub	COG4672 (2.6E-159), pfam05100 (1.2E-130)	Baseplate of native GTA particle, Rcc01696, g13 [*Rhodobacter capsulatus*]	100.0	1.2E-27
							Tail tip protein L [*Escherichia* phage lambda]	100	2.1E-38
**19**	+	14276	14875	199	peptidase	cd08073 (6.7E-26)	Tail tip assembly protein K [*Escherichia* phage lambda]	100.0	3.5E-26

**20**	+	14773	15444	223	megatron	COG4723 (8.1E-102)	Tail tip assembly protein I [*Escherichia* phage lambda]	100	9.4E-39
							Baseplate of native GTA particle, Rcc01698, g15 [*Rhodobacter capsulatus*]	95.7	0.2
**21**	+	15505	18903	1132	megatron	COG4733 (0), pfam13550 (7.1E-42)	Probable baseplate hub protein [*Escherichia* phage T5]	100	1.7E-39
							Baseplate of native GTA particle, Rcc01698, g15 [*Rhodobacter capsulatus*]	99.6	1.8E-13
22	+	18965	19585	206	outer membrane protein lom	pfam06316 (9.5E-57)	Outer membrane protein lom [*Escherichia* phage lambda]	99.9	3.4E-23
23	+	19650	20855	401	tail fiber protein	pfam08400 (3.1E-73)	Tail fiber protein [*Escherichia* phage lambda]	99.5	9.8E-09
24	-	20147	20767	206					
25	+	21029	21973	314	tail Collar	pfam07484 (7.8E-12)			
26	+	21973	22557	194	tail fiber assembly protein	pfam02413 (2.7E-50)			
27	-	22686	23918	410	Protein ea47		Protein ea47 [*Escherichia* phage lambda]	100	3.4E-74
28	-	24509	25399	296	HNH endonuclease	smart00507 (1.7E-05)			
29	-	25396	26973	525	AbiEii toxin, Type IV TA system	pfam13304 (7.8E-11)			
30	-	27812	28882	356	integrase	cd00800 (2.4E-63)			
31	-	28860	29078	72	excisionase	pfam07825 (2.3E-37)			
32	-	29374	29655	93	Protein ea8.5		Protein ea8.5 [*Escherichia* phage lambda]	100.0	1.3E-28
33	-	29847	30395	182	Ea22 early protein	pfam13935 (2.0E-27)			
34	-	30839	31024	61					
35	-	31005	31196	63	DUF1382 domain containling protein	pfam07131 (4.8E-24)			

36	-	31169	31351	60	DUF1317 domain containling protein	pfam07026 (3.0E-39)			
37	-	31348	32028	226	exonuclease	cd22343 (3.0E-41)			
38	-	32025	32810	261	RecT family DNA single-strand annealing protein (SSAP)	TIGR01913 (7.3E-70)			
39	-	32816	33232	138	host-nuclease inhibitor protein Gam	pfam06064 (1.7E-63)			
40	-	33187	33330	47	Kil protein	pfam06301 (5.0E-19)			
41	-	33299	33463	54	protease inhibitor CIII	pfam02061 (5.9E-26)	Protease inhibitor III [*Escherichia* phage lambda]	100.0	3.4E-32
42	-	33536	33904	122	single-stranded DNA binding protein Protein ea10	pfam10800 (1.3E-50)	Protein ea10 [*Escherichia* phage lambda]	100	1.3E-41
43	-	34087	34287	66	antirestriction protein Ral	pfam11058 (2.4E-42)			
44	-	34271	34357	28					
45	-	35037	35438	133	6-mer N-terminal peptide of the N protein (N36)	pfam11438 (8.6E-15)			
46	-	35825	36259	144	membrane-anchored ion channel RexB	pfam15968 (2.9E-74)			
47	-	36275	37114	279	intracellular sensor RexA (Abi component)	pfam15969 (2.5E-115)			
48	-	37227	37940	237	LexA repressor	COG1974 (4.3E-57)	Repressor protein CI [*Escherichia* phage lambda]	100.0	2.1E-28
49	+	38041	38241	66	antirepressor Cro	pfam09048 (4.6E-32)			
50	+	38360	38653	97	CII repressor	pfam05269 (2.4E-38)			
51	+	38686	39585	299	replication protein O	TIGR01610 (1.6E-47)			
52	+	39582	40283	233	replication protein P	pfam06992 (2.5E-84)			
53	+	40280	40570	96	protein ren		Protein ren [*Escherichia* phage lambda]	99.3	3.1E-09

54	+	40644	41084	146	NinB protein	pfam05772 (1.4E-60)			
55	+	41081	41953	290	phosphoadenosine phosphosulphate (PAPS) reductase	cd01713 (3.5E-31)			
56	+	41950	42123	57	NinD	pfam17466 (1.9E-34)	Protein ninD [*Salmonella* phage P22]	100	9.2E-39
57	+	42090	42272	60	NinE	pfam05322 (1.1E-26)			
58	+	42269	42439	56	NinF	pfam05810 (2.1E-22)			
59	+	42429	43043	204	NinG	pfam05766 (7.9E-94)			
60	+	43040	43246	68	NinH	pfam06322 (1.1E-36)			
61	+	43224	43889	221	Ser/Thr Protein Phosphatase	cd07424 (8.2E-118)	Ser/Thr Protein Phosphatase [*Escherichia* virus lambda]	100	2.6E-32
62	+	43886	44509	207	antitermination protein	pfam03589 (1.2E-32)			
63	+	44621	44815	64					
64	+	45186	45509	107	holin	TIGR01594 (2.5E-40)			
65	+	45493	45969	158	lysozyme	COG4678 (1.9E-83)			
66	+	45966	46427	153	Rz lysis protein	pfam03245 (1.3E-48)			
***Roseobacter* phage RDJLφ1**									
1	+	1	432	143	spanin, inner membrane subunit		Spanin, inner membrane subunit [Enterobacteria phage T4]	99.5	7.6E-12
2	+	413	688	91	spanin, outer lipoprotein subunit		Spanin, outer lipoprotein subunit [Enterobacteria phage T4]	99.7	1.5E-16
3	+	693	1013	106	holin-like protein	PHA00649 (3.9E-08)	Holin-like protein 24.1 [*Bacillus* phage SPP1]	88.2	9.2
4	+	1058	1987	309	DNA double-strand break repair protein mre11		DNA double-strand break repair protein mre11 [*Methanocaldococcus jannaschii*]	98.7	6.5E-08
5	+	1977	2330	117	Phosphodiesterase		Phosphodiesterase [*Methanocaldococcus jannaschii*]	97.9	6.8E-04

6	+	2625	2888	87				
7	-	3009	4769	586	ribonucleotide reductase	cd02888 (1.8E-163)		
8	-	4853	5140	95	thiamin biosynthesis sulfur carrier protein ThiS		Thiamin biosynthesis sulfur carrier protein ThiS [*Escherichia coli*]	93.8	0.4
9	-	5137	5310	57				
10	-	5298	5534	78				
11	-	5544	6194	216				
12	+	6275	6610	111				
13	+	6610	6885	91				
14	-	6886	7350	154	virus-type replication-repair nuclease (VRR-Nuc)		Virus-type replication-repair nuclease (VRR-Nuc) [*Salmonella* phage SETP3]	99.7	1.7E-16
15	-	7325	7840	171	dihydrofolate reductase	COG0262 (3.8E-04)			
16	-	7831	8295	154	deoxycytidylate deaminase	pfam00383 (2.8E-24)			
17	-	8292	9179	295	thymidylate synthase	cd00351 (8.7E-34)			
18	-	9169	10215	348	Cof hydrolase/nucleoside triphosphate pyrophosphohydrolase	cd11545 (7.3E-21)			
19	-	10314	11075	253	transcriptional regulator		Putative transcriptional regulator TM1602 [*Thermotoga maritima*]	94.7	0.3
20	-	11068	11268	66					
21	-	11362	11664	100	RNA polymerase sigma factor 54		RNA polymerase sigma factor 54 [*Aquifex aeolicus*]	95.6	0.1
22	-	11763	13301	512	helicase	cd18793 (5.6E-08)			
23	-	13305	13664	119	5'-deoxynucleotidase YfbR		5'-deoxynucleotidase YfbR [*Escherichia coli* K-12]	97.8	1.7E-04
24	-	13654	13899	81	5'-deoxynucleotidase YfbR		5'-deoxynucleotidase YfbR-like [*Agrobacterium fabrum* str. C58]	99.9	2.1E-21

25	-	13911	14294	127					
26	-	14364	14990	208	DNA topoisomerase IV, alpha subunit		DNA topoisomerase IV, alpha subunit [*Methanococcus jannaschii*]	99.4	1.2E-12
27	+	14993	15313	106					
28	-	15350	17866	838	DNA polymerase A domain protein	cd08642 (5.3E-66)			
29	-	17870	18106	78			winged-helix like protein with metal binding site [*Pyrococcus furiosus* DSM 3638]	93.2	0.4
30	-	18123	18374	83			Uncharacterized protein conserved in bacteria (DUF2312) [*Bosea* sp. Root381]	98.3	8.8E-06
31	-	18371	18676	101			endopeptidase domain like containing protein [*Pseudomonas aeruginosa* PAO1]	91.1	0.4
32	-	18802	19833	343	replication factor C	pfam13191 (1.9E-04)	Replication factor C3 [*Saccharomyces cerevisiae*]	99.6	5.6E-13
33	+	20003	20305	100					
34	-	20391	21032	213	single-stranded DNA-binding protein	pfam10991 (3.3E-31)	Single-stranded DNA-binding protein [Enterobacter phage Enc34]	100.0	1.1E-28
35	-	21173	21700	175	RNase T-like exonuclease	pfam16473 (3.9E-33)			
36	-	21700	21921	73					
37	-	21915	22370	151	transcriptional regulator CtrA	cd00383 (6.4E-16)	response regulator protein mtrA [*Mycobacterium Tuberculosis*]	98.6	1.6E-05
38	-	22762	23694	310	chromosomal replication initiator protein dnaA		Chromosomal replication initiation factor DnaA [*Aquifex aeolicus*]	97.4	1.3E-03
39	-	23706	24041	111	DUF3268 family protein	pfam11672 (6.7E-18)			
40	-	24034	24288	84					
41	-	24288	24569	93					

42	-	24566	26008	480	CRISPR/Cas system-associated protein Cas4	pfam10926 (5.8E-52)			
43	-	26009	26269	86					
44	-	26269	26478	69					
45	-	26471	26686	71	D-lysine 5,6-aminomutase beta subunit KamE		D-lysine 5,6-aminomutase beta subunit KamE [*Clostridium sticklandii*]	91.8	1.1
46	-	27121	27597	158	membrane spanning protein TolA		Putative protein p53 [Bacteriophage APSE-1]	99.0	3.1E-09
47	+	27764	30172	802	bifunctional DNA primase/polymerase	cd04859 (2.0E-16)			
48	+	30234	30452	72					
49	+	30592	30843	83					
50	+	30840	31205	121	chromodomain-helicase-DNA-binding protein 1-like	PHA00684 (1.8E-27)	Chromodomain-helicase-DNA-binding protein 1-like [*Homo sapiens*]	99.7	1.2E-15
51	+	31198	31581	127	DNA ligase		Adenylation domain of NAD^{+}-dependent DNA ligase [*Thermus filiformis*]	99.2	9.9E-11
52	+	31699	32037	112					
53	+	32809	33309	166					
54	+	33491	33895	134					
55	+	34046	34588	180	uracil DNA glycosylase	pfam07799 (9.6E-45)			
56	+	34895	35275	126	pyrimidine dimer DNA glycosylase	pfam03013 (8.6E-14)			
57	+	35586	35855	89					
58	+	35840	36022	60					
59	+	36080	36388	102	fucose-specific lectin		Fungal fucose-specific lectin [*Ralstonia solanacearum*]	92.2	1.0

60	+	36407	36904	165					
61	+	36921	37580	219	endolysin	pfam01471 (2.0E-07)	L-alanyl-D-glutamate peptidase Ply [*Listeria* phage A500]	99.3	2.6E-11
**62**	+	37752	38327	191	terminase small subunit		Terminase small subunit [*Lactococcus* phage SK1]	99.4	9.2E-12
**63**	+	38331	39689	452	terminase large subunit	TIGR01547 (5.6E-35)	Terminase, large subunit [*Bacillus* phage SPP1]	100	3.0E-39
							Terminase, large subunit [*Escherichia* virus HK97]	100	3.4E-31
**64**	-	39979	40596	205					
**65**	-	40659	40952	97					
**66**	-	40981	41193	70					
**67**	+	41363	42802	479	portal		Portal protein [*Thermus* phage P7426]	100	1.6E-36
							Phage portal protein Rcc01684, HK97 family, g3 [*Rhodobacter capsulatus*]	100.0	2.2E-26
**68**	+	42802	43398	198	adaptor		Head completion protein gp15 [*Bacillus* phage SPP1]	96.2	0.3
							Adaptor protein Rcc01688, g6 [*Rhodobacter capsulatus*]	94.2	0.2
**69**	+	43411	43785	124	stopper		Head-tail connector protein FII [*Escherichia* phage lambda]	98.9	1.0E-07
							Stopper protein Rcc01689, g7 [*Rhodobacter capsulatus*]	92.2	3
**70**	+	43786	44760	324	head morphogenesis protein	pfam04233 (2.0E-06)	Minor head protein GP7 [*Bacillus* phage SPP1]	99.9	4.1E-24
							Putative capsid assembly protein F [*Escherichia* phage Mu]	99.7	1.5E-16
**71**	+	44852	45250	132	prohead protease	pfam14550 (3.6E-11)	Prohead protease [Enterobacteria phage HK97]	99.6	8.9E-14
**72**	+	45252	46364	370		NF033804 (1.8E-03)			
**73**	+	46442	46867	141	capsid fiber protein		Capsid fiber protein [*Bacillus* phage phi29]	99.8	6.0E-17

							Capsid Stabilizing Protein [*Pseudoalteromonas* phage TW1]	98.5	1.8E-05
**74**	+	46906	47940	344	major capsid	pfam03864 (8.1E-05)	Major capsid protein [*Escherichia* phage lambda]	99.9	1.6E-22
							Major capsid protein [Enterobacteria phage HK97]	98.1	1.6E-04
							Major capsid protein Rcc01687, g5 [*Rhodobacter capsulatus* DE442]	98.0	9.1E-04
**75**	+	48014	48679	221					
**76**	+	48682	49092	136	tail completion protein		Putative capsid assembly protein G [*Escherichia* phage Mu]	98.4	2.3E-06
							Tail completion protein S [*Escherichia* phage P2]	96.3	2.7E-02
**77**	+	49089	49505	138	tail terminator		Tail completion protein gp17 [*Bacillus* phage SPP1]	98.1	6.0E-04
							Tail terminator protein Rcc01690, g8 [*Rhodobacter capsulatus*]	97.6	1.2E-02
**78**	+	49523	51043	506	tail tube	pfam18906 (7.0E-11)	Tail tube protein [*Escherichia* phage T5]	99.8	4.8E-16
							Phage major tail protein, TP901-1 family, Rcc01691, g9 [*Rhodobacter capsulatus*]	94.0	3.1
**79**	+	51129	51575	148	tail assembly chaperone		Tail assembly chaperone [Bacteriophage HK97]	94.7	0.4
**80**	+	51941	55030	1029	tail tape measure protein	TIGR01760 (1.5E-46)	Probable tape measure protein [*Bacillus* phage Spbeta]	99.9	5.0E-17
**81**	+	55031	55651	206	distal tail	pfam09343 (2.1E-62), TIGR02217 (9.0E-54)	Baseplate of native GTA particle, Rcc01695, g12 [*Rhodobacter capsulatus*]	100	8.2E-50
							Tail tip protein M [*Escherichia* phage lambda]	99.5	2.3E-13
**82**	+	55648	56553	301	hub	pfam09931 (9.6E-75), pfam09356 (2.4E-33)	Baseplate of native GTA particle, Rcc01696, g13 [*Rhodobacter capsulatus*]	100	6.4E-53
							Tail tip protein L [*Escherichia* phage lambda]	100.0	5.2E-27

**83**	+	56522	56971	149	phage cell wall peptidase	COG0791 (1.4E-18), TIGR02219 (3.6E-51)	Tail tip assembly protein K [*Escherichia* phage lambda]	99.0	3.9E-08
							NlpC/P60 family protein [*Trichormus variabilis* ATCC 29413]	99.0	9.2E-08
**84**	+	56975	61060	1361	megatron	cd19607 (2.3E-139), pfam13547 (8.5E-155)	Baseplate of native GTA particle, Rcc01698, g15 [*Rhodobacter capsulatus*]	100	6.0E-206
							Tip attachment protein J [*Escherichia* phage lambda]	99.1	2.0E-09
85	+	61070	62152	360	tail fiber protein	pfam10983 (1.0E-32)	Cement (decoration) protein [cyanophage Pam1]	92.2	1.1
86	+	62161	62400	79			no hit		
87	+	62448	62648	66			no hit		

[a]ORFs in the genomic region that corresponds to the RcGTA head-tail cluster are indicated in bold.

[b]Putative functions are predicted based on the function of homologs from the Conserved Domain Database or detected by HHpred search against the PDB, SCOPe, and UniprotKb/Swiss-Prot databases.

[c]Significant hits of each ORF in the Conserved Domain Database and their e-values.

[d]HHpred search against the PDB, SCOPe, and UniprotKb/Swiss-Prot databases were conducted on ORFs without conserved domain or in the genomic regions mainly encoding structural proteins.

[e]Homologs belonging to the RcGTA proteins were indicated in purple.

Table S3. vB_MseS-P1 and four reference phage genes that are homologous to the *Methylobacterium nodulans* ORS 2060 LC1–LC5 genes and RcGTA-like cluster genes.

ORF	aa length	Putative function	RcGTA-like homologs	*Methylobacterium nodulans* ORS 2060					RcGTA	Top hit of the other RcGTA-like gene cluster (aa identity (%))	Order of the top hit bacterial strain
				LC1	LC2	LC3	LC4	LC5			
**vB_MseS-P1**											
31	570	terminase large subunit						19.6			
32	411	portal protein	g3	27.3	27.4	27.4		30.2	22.2	*Methylocystis* sp. SC2 (24.9)	*Rhizobiales*
33	229	prohead protease	g4		30.8	30.8			51.1	*Hyphomicrobium nitrativorans* NL23 (43.0)	*Rhizobiales*
34	448	major capsid protein		26.6							
36	186	adaptor	g6	36.6				29.1		*Hyphomicrobium denitrificans* ATCC 51888 (29.2)	*Rhizobiales*
37	117	stopper			48.6	48.6					
38	147	tail completion protein			36.1	37.3					
39	128	tail terminator	g8		36.2	43.6				*Hyphomicrobium denitrificans* ATCC 51888 (32.5)	*Rhizobiales*
40	151	tail tube protein	g9	27.8	31.3	32.0	32.2	25.9		*Ketogulonicigenium vulgare* Y25 (32.4)	*Rhodobacterales*
41	141	tail assembly protein			36.3	36.3					
44	724	tail tape measure protein			32.4	32.4	30.2				
55	416	tail fiber protein	*rcc00171*						29.9		
***Rhizobium* phage 16-3**											
2	525	terminase large subunit		24.2	26.2	26.0		27.0			
3	396	portal protein	g3	27.7	22.5	22.5		25.6		*Parvularcula bermudensis* HTCC2503 (22.4)	*Parvularculales*
4	214	prohead protease	g4						29.5	*Rhodopseudomonas palustris* BisB18 (28.2)	*Rhizobiales*

6	388	major capsid protein	g5					34.2	31.2	*Nitrobacter hamburgensis* X14 (25.6)	*Rhizobiales*
9	181	adaptor	g6							*Parvibaculum lavamentivorans* DS-1 (34.8)	*Rhizobiales*
11	115	stopper			41.8	41.8					
12	138	tail completion protein		29.3	31.7	33.1					
13	135	tail terminator	g8		35.6	42.9				*Hyphomicrobium denitrificans* ATCC 51888 (36.0)	*Rhizobiales*
15	148	tail tube protein			34.3	34.3	30.3				
16	117	tail assembly protein		38.5	38.2	38.2		30.2			
18	859	tail tape measure protein			28.8	29.7	32.4				
***Escherichia* phage HK97**											
2	504	terminase large subunit		24.2	24.1	25.2		24.3			
3	424	portal protein	g3	29.5	22.7	22.7		30.2	26.2	*Methylocystis* sp. SC2 (26.2)	*Rhizobiales*
4	225	prohead protease	g4						32.0	*Hyphomicrobium denitrificans* ATCC 51888 (39.4)	*Rhizobiales*
5	385	major capsid protein	g5							*Methylobacterium populi* BJ001 (23.8)	*Rhizobiales*
7	112	stopper	g7							*Agrobacterium fabrum* strain 1D132 (27.9)	*Rhizobiales*
14	1089	tail tape measure protein	g11						43.3	*Dinoroseobacter shibae* DFL 12 (46.3)	*Rhodobacterales*
***Escherichia* phage Lambda**											
16	853	tail tape measure protein		33.1	32.5						
19	199	peptidase	g14							*Xanthobacter autotrophicus* Py2 (33.1)	
***Roseobacter* phage RDJLφ1**											
80	1029	tail tape measure protein	g11							*Octadecabacter antarcticus* 307 (61.0)	*Rhodobacterales*
81	206	distal tail	g12	38.2	38.2	38.6	38.5	38.2	45.2	*Parvibaculum lavamentivorans* DS-1 (46.7)	*Rhizobiales*

82	301	hub	g13	43.0	43.0	42.8	43.0	43.0	39.5	*Hyphomicrobium denitrificans* ATCC 51888 (50.7)	*Rhizobiales*
83	149	peptidase	g14	47.0	47.7	46.3	47.7	47.7	49.7	*Caulobacter crescentus* NA1000 (53.3)	*Caulobacterales*
84	1361	megatron	g15	39.2	40.0	39.6	39.4	39.3	35.9	*Hyphomicrobium nitrativorans* NL23 (38.8)	*Rhizobiales*
85	360	tail fiber protein	*rcc00171*	31.4	31.5	29.5	31.4	31.4	33.3	*Methylobacterium extorquens* DM4 (34.5)	*Rhizobiales*

Table S4. Annotation of predicted ORFs in the *Methylobacterium nodulans* ORS 2060 genome segments of LC1–LC5-associated putative prophages or prophage remnants and their homologs in the Phage Orthologous Group (POG) database (1).

ORF[a]	Strand	Left	Right	aa length	Putative function[b]	Top POG hit (E-value)[c]	Conserved domain (E-value)[d]	HHpred search[e] Significant Hits[f]	Probability (%)	E-value
**LC1-associated prophage**										
1	-	2076723	2077784	353	integrase	gi|168495175|ref|YP_001686913.1| (3.7E-16)	cd00796 (2.5E-18)			
2	-	2077756	2078031	91	excisionase			Excisionase [*Shigella* phage SfV]	98.8	2.2E-08
3	-	2078019	2078324	101						
4	-	2078321	2078800	159						
5	-	2078797	2079168	123						
6	-	2079165	2080514	449						
7	-	2080511	2081566	351						
8	-	2081832	2082224	130						
9	-	2082228	2082998	256	regulatory protein Rha	lcl|315274295-1| (1.4E-22)	pfam09669 (6.0E-29)			
10	-	2083002	2083094	30						
11	-	2083180	2083863	227	transcriptional regulator, XRE family	gi|41057317|ref|NP_958215.1| (2.0E-08)	COG1396 (4.6E-11)			
12	+	2083947	2084198	83	YdaS antitoxin of toxin-antitoxin system, YdaS/YdaT	gi|327198607|ref|YP_004327351.1| (1.3E-08)	pfam15943 (5.5E-26)			
13	+	2084449	2084871	140						
14	+	2084868	2085170	100						
15	+	2085170	2085553	127	virus-type replication repair nuclease (VRR-NUC)	gi|146329976|ref|YP_001210284.1| (2.3E-06)	smart00990 (2.4E-10)			

16	+	2085556	2086005	149						
17	+	2086005	2086325	106	septation protein SpoVG			Putative septation protein SpoVG [*Bacillus subtilis*]	99.9	1.1E-21
18	+	2086322	2086927	201	bifunctional DNA primase/polymerase	lcl|168229312-1| (2.6E-12)	smart00943 (1.5E-31)			
19	+	2086977	2088506	509	virulence-associated protein E	lcl|209363581-3| (8.2E-52)	pfam05272 (6.9E-105)			
20	+	2088646	2088894	82						
21	+	2088891	2089037	48	ROS/MUCR transcriptional regulator		pfam05443 (3.8E-05)			
22	+	2089091	2089735	214	transcription termination/antitermination factor NusG		pfam02357 (2.3E-08)			
23	+	2091066	2092379	437	oxalate/formate antiporter		TIGR04259 (0)			
24	-	2093023	2093298	91						
25	+	2093781	2094530	249	cAMP-binding protein-catabolite gene activator		COG0664 (1.31E-29)			
26	+	2094530	2094967	145	5-methylcytosine-specific restriction endonuclease McrA	gi|84662652|ref|YP_453617.1| (6.2E-21)	COG1403 (4.2E-4)			
**27**	+	2095072	2095506	144	terminase, small subunit		pfam05119 (8.1E-15)			
**28**	+	2095478	2097313	611	terminase, large subunit	gi|168495132|ref|YP_001686870.1| (0)	COG4626 (5.0E-56)			
**29**	+	2097313	2097492	59	membrane protein			CD9 antigen, membrane protein [*Homo sapiens*]	91.6	3.9
								Membrane protein complex ion-driven motor [*Flavobacterium johnsoniae*]	90.8	1.6

**30**	+	2097492	2098913	473	portal protein	gi|313575378|ref|CBR26907.1| (5.0E-84)	pfam04860 (1.4E-93)	Portal protein [*Enterobacteria* phage HK97]	100	1.3E-39
								Phage portal protein Rcc01684, HK97 family, g3 [*Rhodobacter capsulatus*]	100	2.7E-38
**31**	+	2098910	2099818	302	capsid assembly protease	gi|33770512|ref|NP_892049.1| (7.4E-37)	cd07022 (7.3E-85)	Capsid assembly protease C [*Escherichia* phage lambda]	100	1.3E-39
**32**	+	2100063	2101379	438	major capsid protein	gi|313575380|ref|CBR26909.1| (1.1E-25)	TIGR01554 (2.7E-80)	Major capsid protein [*Enterobacteria* phage HK97]	100	4.3E-36
								Major capsid protein Rcc01687, g5 [*Rhodobacter capsulatus* DE442]	100	6.1E-31
**33**	+	2101442	2101831	129	cementing protein			Cementing protein [*Bordetella* phage BPP-1]	98.1	7.4E-05
**34**	+	2101897	2102367	156						
**35**	+	2102394	2102882	162	adaptor protein	gi|238695595|ref|YP_002922622.1| (6.3E-07)	cd08054 (2.1E-22)	Adaptor protein Rcc01688, g6 [*Rhodobacter capsulatus*]	99.9	6.2E-22
**36**	+	2102882	2103211	109	stopper protein		pfam05521 (2.3E-09)	Stopper protein Rcc01689, g7 [*Rhodobacter capsulatus*]	99.9	5.3E-20
								Head completion protein gp16 [*Bacillus* phage SPP1]	99.8	3.9E-19
**37**	+	2103211	2103783	190	tail completion protein		pfam04883 (1.2E-06)	Capsid assembly protein G [*Escherichia* phage Mu]	99.1	8.5E-10

								Tail completion protein S [*Escherichia* phage P2]	98.1	5.6E-05
								Tail completion protein Z [*Escherichia* phage lambda]	92.1	1
**38**	+	2103780	2104229	149	tail terminator protein		pfam11367 (8.8E-20)	Tail terminator protein Rcc01690, g8 [*Rhodobacter capsulatus*]	99.8	1.7E-18
**39**	+	2104226	2104675	149	tail tube protein			Phage major tail protein, TP901-1 family, Rcc01691, g9 [*Rhodobacter capsulatus*]	99.9	5.3E-19
**40**	+	2104672	2105058	128	tail tube protein, GTA-gp10, tail chaperone 1		pfam11836 (4.1E-26)			
**41**	+	2105103	2105231	42						
**42**	+	2105297	2107504	735	tail tape measure protein			Probable tape measure protein [*Bacillus* phage Spbeta]	98.9	1.2E-05
								Tape measure protein [*Escherichia* phage lambda]	97.6	5.4E-01
**43**	+	2107504	2108139	211	distal tail		pfam09343 (2.6E-104), TIGR02217 (2.6E-85)	Baseplate of native GTA particle, Rcc01695, g12 [*Rhodobacter capsulatus*]	100	3.3E-49
**44**	+	2108136	2109020	294	hub	gi|331028136|ref|YP_004421850.1| (6.8E-81)	pfam09931 (2.0E-73), TIGR02218 (3.8E-73)	Baseplate of native GTA particle, Rcc01696, g13 [*Rhodobacter capsulatus*]	100	2.5E-53
**45**	+	2109001	2109453	150	peptidase		TIGR02219 (4.0E-60)			

**46**	+	2109450	2113352	1300	megatron		pfam13547 (0), pfam13550 (6.5E-39)	Baseplate of native GTA particle, Rcc01698, g15 [*Rhodobacter capsulatus*]	100	8.4E-207
								Probable baseplate hub protein [*Escherichia* phage T5]	99.5	6.8E-10
47	+	2113345	2115012	555	tail fiber protein	lcl|331028139-1| (8.6E-19)	pfam10983 (8.1E-34)	L-shaped tail fiber protein [*Escherichia* phage T5]	99.5	1.8E-12
48	+	2115044	2115685	213	lysozyme	lcl|167832390-1| (7.0E-16)	COG3926 (9.8E-61)			
**LC2-associated prophage**										
1	-	2681646	2682287	213	lysozyme	lcl|167832390-1| (3.9E-17)	COG3926 (6.1E-61)			
2	-	2682319	2683983	554	tail fiber protein	lcl|331028139-1| (5.1E-17)	pfam10983 (8.8E-28), cd10144 (2.2E-27)	L- shaped tail fiber [*Escherichia* virus T5]	99.5	1.4E-12
**3**	-	2683976	2687881	1301	megatron		pfam13547 (0), pfam13550 (1.0E-38)	Baseplate of native GTA particle, Rcc01698, g15 [*Rhodobacter capsulatus*]	100	6.1E-207
**4**	-	2687878	2688330	150	peptidase		TIGR02219 (3.7E-61)			
**5**	-	2688311	2689195	294	hub	gi|331028136|ref|YP_004421850.1| (1.7E-81)	TIGR02218 (3.0E-75), pfam09356 (2.8E-39)	Baseplate of native GTA particle, Rcc01696, g13 [*Rhodobacter capsulatus*]	100	8.3E-53
**6**	-	2689192	2689827	211	distal tail		pfam09343 (3.3E-103)	Baseplate of native GTA particle, Rcc01695, g12 [*Rhodobacter capsulatus*]	100	3.1E-49
**7**	-	2689827	2692925	1032	tail tape measure protein	lcl|309386974-2| (1.6E-20)	pfam06791 (7.5E-34)	Tape measure protein [*Escherichia* phage lambda]	100	1.5E-27
**8**	-	2693074	2693484	136	tail tube protein, GTA-gp10		pfam11836 (2.8E-23)			

**9**	-	2693484	2693930	148	tail tube protein		pfam06199 (1.5E-21)	Phage major tail protein, TP901-1 family, Rcc01691, g9 [*Rhodobacter capsulatus*]	99.9	5.9E-19
**10**	-	2693952	2694350	132	tail terminator protein	gi|195546543|ref|YP_002117571.1| (2.4E-16)	pfam11367 (4.8E-25)	Tail terminator protein Rcc01690, g8 [*Rhodobacter capsulatus*]	99.9	1.6E-21
**11**	-	2694347	2694844	165	tail completion protein	gi|195546542|ref|YP_002117570.1| (2.3E-13)	pfam04883 (1.5E-07)	Capsid assembly protein G [*Escherichia* phage Mu]	98.7	5.9E-08
								Tail completion protein S [*Escherichia* phage P2]	97.4	1.3E-03
								Tail completion protein Z [*Escherichia* phage lambda]	95.0	0.5
**12**	-	2694989	2695354	121	stopper protein		pfam05521 (9.1E-15)	Stopper protein Rcc01689, g7 [*Rhodobacter capsulatus*]	99.8	1.7E-19
								Head completion protein gp16 [*Bacillus* phage SPP1]	99.9	8.6E-20
**13**	-	2695351	2695635	94	adaptor protein	gi|209552427|ref|YP_002284342.1| (7.2E-09)	cd08054 (1.8E-20)	Head completion protein gp15 [*Bacillus* phage SPP1]	99.6	4.5E-13
								Adaptor protein Rcc01688, g6 [*Rhodobacter capsulatus*]	99.3	2.2E-10
**14**	-	2695657	2697960	767	LPXTG-anchored collagen-like adhesin Scl2/SclB	gi|20065816|ref|NP_612899.1| (1.8E-22)	NF038329 (5.3E-30)			
**15**	-	2697953	2699317	454	portal protein	gi|157166043|ref|YP_001449295.1| (1.1E-156)	pfam04860 (1.2E-82)	Portal protein [*Streptomyces* phage phiC31]	100	3.3E-42

							Phage portal protein Rcc01684, HK97 family, g3 [*Rhodobacter capsulatus*]	100	1.7E-41
**16**	-	2699324	2699623	99					
**17**	-	2699679	2701664	661	major capsid/prohead protease, HK97 family	lcl|209552422-3| (2.2E-168)	TIGR01554 (1.1E-19), TIGR01543 (7.8E-09)	Capsid polyprotein [*Pseudomonas* phage PAJU2]	100	1.5E-60
								Major capsid protein Rcc01687, g5 [*Rhodobacter capsulatus* DE442]	99.9	1.5E-24
**18**	-	2701679	2703499	606	terminase, large subunit	gi|168495132|ref|YP_001686870.1| (4.0E-177)	COG4626 (1.9E-58)			
**19**	-	2703462	2703893	143	terminase, small subunit		pfam05119 (9.1E-22)			
20	-	2704030	2704302	90						
21	-	2704306	2704668	120	HNH endonuclease	gi|32128472|ref|NP_859007.1| (8.6E-19)		HNH endonuclease [*Geobacillus* virus E2]	98.4	1.4E-06
22	-	2704661	2704801	46						
23	-	2705238	2705828	196	transcription termination/antitermination factor NusG		cd09892 (6.9E-12)			
24	-	2705828	2706250	140	MucR family transcriptional regulator		pfam05443 (3.7E-67)			
25	-	2706577	2709489	970	bifunctional DNA primase/polymerase	lcl|195546631-1| (4.3E-16)	pfam09250 (1.1E-40)			
26	-	2709486	2710607	373	exonuclease V	gi|162135088|ref|YP_001595829.1| (1.4E-104)	COG0507 (6.8E-34)			

27	-	2710604	2710924	106	septation protein SpoVG			Putative septation protein SpoVG [*Bacillus subtilis*]	99.9	7.6E-22
28	-	2710921	2711250	109						
29	-	2711253	2711936	227	modification methylase	gi|294338273|ref|CBJ94311.1| (9.0E-14)		Modification methylase [*Paramecium bursaria* Chlorella virus XZ-6E]	99.3	1.1E-10
30	-	2711933	2712367	144						
31	-	2712370	2712759	129	virus-type replication repair nuclease (VRR-NUC)	gi|62327129|ref|YP_223917.1| (2.9E-07)	smart00990 (2.9E-14)			
32	+	2712972	2713688	238						
33	-	2713811	2714191	126						
34	-	2714188	2714349	53						
35	-	2714346	2714645	99	repressor protein C2			Repressor protein C2 [*Enterobacteria* phage P22]	93.9	1.1
36	+	2715178	2715558	126	LexA repressor		cd06529 (1.2E-4)			
37	+	2715631	2715942	103						
38	+	2715942	2716355	137						
39	+	2716355	2716636	93						
40	+	2716630	2716938	102						
41	+	2716941	2717753	270						
42	+	2717750	2718061	103						
43	+	2718058	2719008	316	YqaJ-like viral recombinase	lcl|238801623-1| (3.1E-18)	pfam09588 (4.4E-15)			
44	+	2719005	2720009	334	RecT family DNA single-strand annealing protein (SSAP)	lcl|89152454-2| (1.3E-35)	pfam03837 (6.9E-12)			
45	+	2720013	2720498	161						

46	+	2720509	2720955	148	two-component system response regulator			two-component system response regulator [*Streptomyces coelicolor* A3(2)]	91.0	5.5
47	+	2720958	2721377	139						
48	-	2721468	2721761	97	cellulose synthase			Cellulose synthase 1 [*Gluconacetobacter xylinus*]	99.2	9.6E-10
49	-	2721927	2722178	83	magnetosome protein MamM, cation efflux protein family			Magnetosome protein MamM, Cation efflux protein family [*Magnetospirillum gryphiswaldense*]	95.6	0.4
50	+	2722307	2723200	297	site-specific DNA-adenine methylase	gi|56692911|ref|YP_164275.1| (3.9E-89)	COG0338 (1.6E-48)			
51	-	2723293	2723544	83	magnetosome protein MamM, cation efflux protein family			Magnetosome protein MamM, Cation efflux protein family [*Magnetospirillum gryphiswaldense*]	91.4	3.2
52	+	2723622	2724020	132						
53	+	2724135	2724638	167	nucleoside 2-deoxyribosyltransferase			Nucleoside 2-deoxyribosyltransferase [*Lactobacillus leichmannii*]	99.7	1.4E-14
54	+	2724641	2725000	119						
55	+	2724997	2725395	132	DNA gyrase inhibitor YacG			DNA gyrase inhibitor YacG [*Escherichia coli* K-12]	96.2	1.4E-02
56	+	2725398	2725772	124	excisionase			Excisionase [*Shigella* phage SfV]	99.1	2.3E-09

57	+	2725765	2727030	421	integrase	gi|168495175|ref|YP_001686913.1| (2.2E-13)	cd00800 (1.7E-10)			
**LC5-associated prophage**										
1	-	7402951	7403526	191	lysozyme	lcl|45580747-1| (9.2E-17)	COG3926 (4.8E-55)			
2	-	7403601	7405268	555	tail fiber protein	lcl|331028139-1| (7.8E-19)	pfam10983 (6.1E-34), cd10144 (4.1E-31)	L-shaped tail fiber protein [*Escherichia* virus T5]	99.6	8.1E-14
**3**	-	7405261	7409163	1300	megatron		pfam13547 (0), cd19607 (1.2E-164)	Baseplate of native GTA particle, Rcc01698, g15 [*Rhodobacter capsulatus*]	100	1.2E-200
**4**	-	7409160	7409612	150	peptidase		TIGR02219 (2.0E-61)			
**5**	-	7409593	7410477	294	hub	gi|331028136|ref|YP_004421850.1| (3.2E-81)	pfam09931 (5.4E-73), TIGR02218 (2.4E-72)	Baseplate of native GTA particle, Rcc01696, g13 [*Rhodobacter capsulatus*]	100	3.7E-53
**6**	-	7410474	7411109	211	distal tail		pfam09343 (2.7E-104)	Baseplate of native GTA particle, Rcc01695, g12 [*Rhodobacter capsulatus*]	100	2.0E-48
**7**	-	7411109	7413133	674	tail tape measure protein			Tape measure protein [*Escherichia* phage T1]	98.2	1.6E-04
**8**	-	7413137	7413292	51	tail assembly protein			Tail assembly protein E [*Escherichia* phage P2]	94.4	0.2
**9**	-	7413292	7413687	131	tail tube protein, GTA-gp10		pfam11836 (3.1E-23)			
**10**	-	7413706	7414155	149	tail tube		COG5437 (7.6E-4)	Phage major tail protein, TP901-1 family, Rcc01691, g9 [*Rhodobacter capsulatus*]	99.9	9.4E-19

**11**	+	7414531	7415040	169	cAMP-dependent protein kinase		cd00038 (6.9E-27)	cGMP-dependent protein kinase [*Plasmodium vivax* Sal-1]	99.8	2.1E-17
**12**	-	7415224	7415634	136	tail terminator protein	gi|66394699|ref|YP_240829.1| (3.0E-07)	pfam11367 (2.5E-20)	Tail terminator protein Rcc01690, g8 [*Rhodobacter capsulatus*]	99.9	2.5E-20
**13**	-	7415635	7416189	184	tail completion protein			Putative capsid assembly protein G [*Escherichia* phage Mu]	99.1	9.0E-10
								Tail completion protein S [*Escherichia* phage P2]	98.0	2.5E-04
								Tail completion protein Z [*Escherichia* phage lambda]	91.6	1.1
**14**	-	7416186	7416524	112	stopper protein		pfam05521 (1.8E-07)	Stopper protein Rcc01689, g7 [*Rhodobacter capsulatus*]	99.9	1.2E-20
**15**	+	7416659	7416787	42						
**16**	+	7416830	7417138	102	toxin component HigB of the HigAB toxin-antitoxin system		pfam09907 (9.1E-29)			
**17**	+	7417148	7417525	125	antitoxin component HigA of the HigAB toxin-antitoxin system		COG5499 (1.4E-36)			
**18**	-	7417605	7418210	201	adaptor protein		TIGR02215 (4.1E-12), cd08054 (4.5E-09)	Adaptor protein Rcc01688, g6 [*Rhodobacter capsulatus*]	100.0	1.1E-27
**19**	-	7418213	7418506	97	DNA-packaging protein FI			DNA-packaging protein FI [*Enterobacteria* phage lambda]	92.4	0.5

**20**	-	7418581	7419834	417	major capsid protein	gi|255033737|ref|YP_003090181.1| (1.1E-89)	TIGR01554 (2.7E-110)	Major capsid protein [*Enterobacteria* phage HK97]	100	2.5E-37
								Major capsid protein Rcc01687, g5 [*Rhodobacter capsulatus* DE442]	100	2.6E-34
**21**	-	7419899	7420612	237	caseinolytic protease (ClpP)	gi|255033736|ref|YP_003090180.1| (1.9E-69)	cd07016 (1.3E-70)	ATP-dependent Clp protease proteolytic subunit [*Chlamydomonas reinhardtii*]	99.9	1.6E-20
**22**	-	7420584	7421870	428	portal protein	gi|313575378|ref|CBR26907.1| (3.1E-49)	pfam04860 (5.0E-87)	Portal protein [*Enterobacteria* phage HK97]	100	3.5E-41
								Phage portal protein Rcc01684, HK97 family, g3 [*Rhodobacter capsulatus*]	100	5.6E-40
**23**	-	7421867	7423612	581	terminase, large subunit	gi|18249900|ref|NP_543088.1| (0)	COG4626 (7.5E-124)	Putative terminase large subunit [*Shigella* phage SfV]	100	4.5E-63
**24**	-	7423617	7424075	152	terminase, small subunit	gi|148747728|ref|YP_001285807.1| (3.7E-08)	pfam05119 (4.9E-19)	Terminase small subunit [*Enterobacteria* phage HK97]	99.7	4.5E-17
25	-	7424176	7424463	95						
26	-	7425405	7425620	71						
27	-	7425859	7426062	67	stress response protein CsbD		COG3237 (1.2E-22)			
28	-	7426078	7426401	107						

29	-	7426410	7426637	75	tail-anchored protein insertion receptor			Tail-anchored protein insertion receptor WRB [*Homo sapiens*]	91.7	2.6
30	-	7426630	7426881	83						
31	-	7426878	7427156	92						
32	-	7427153	7428040	295	resolvase	lcl|216905985-1| (8.1E-37)	smart00857 (5.3E-38)			
33	-	7428120	7428392	90						
34	+	7428650	7430497	615	$Ca2^{+}$-binding protein, RTX toxin-related		COG2931 (3.8E-19)			
35	-	7430662	7431519	285	IS5 family transposase		NF033580 (4.6E-61)			
36	-	7431685	7432455	256						
37	-	7432617	7433075	152	RNA polymerase sigma factor			RNA polymerase sigma GP34 factor [*Bacillus* phage SP01]	95.0	0.3
38	+	7433186	7433467	93	ribonuclease toxin, BrnT, of type II toxin-antitoxin system		pfam04365 (3.7E-27)			
39	+	7433454	7433816	120	transcriptional regulator YiaG, XRE family		COG2944 (2.3E-15)			
40	-	7434437	7437403	988	TPR (tetratricopeptide repeats)-repeat lipoprotein		TIGR02917 (1.3E-51)			
41	-	7437607	7438542	311	DNA-binding transcriptional regulator YhcF, GntR family	lcl|38707961-1| (1.2E-07)	pfam13730 (2.2E-10)	Replication protein repL [*Escherichia* phage P1]	98.6	1.9E-06
42	-	7438854	7439246	130	N6-adenosine-specific RNA methylase IME4	gi|89152451|ref|YP_512288.1| (8.6E-06)	COG4725 (2.2E-16)			
43	-	7439784	7439993	69						
44	-	7439993	7440181	62						

45	-	7440178	7440435	85					
46	+	7440500	7440754	84	CII repressor			P22 C2 repressor [*Salmonella* bacteriophage P22]	97.9	1.5E-04
47	+	7440774	7440977	67					
48	+	7440986	7442020	344	integrase	gi|168495175|ref|YP_001686913.1| (1.6E-18)	cd00800 (3.2E-05)			
**LC3-associated prophage remnants**									
1	-	4312755	4313645	296	transcriptional regulator, LysR family		cd08421 (2.2E-87)			
2	+	4313759	4313983	74						
3	+	4314028	4314783	251	sulfite exporter TauE/SafE		pfam01925 (2.2E-13)			
4	-	4315345	4315950	201	TolA protein			TolA protein [*Vibrio cholerae*]	92.4	0.9
5	+	4316816	4317508	230	transcription termination/antitermination factor NusG		COG0250 (1.9E-24)			
6	-	4317670	4317999	109						
7	-	4318083	4318331	82						
8	+	4318714	4318854	46						
9	+	4318847	4319209	120	HNH endonuclease	gi|32128472|ref|NP_859007.1| (8.6E-19)		HNH endonuclease [*Geobacillus* virus E2]	98.4	1.4E-06
10	+	4319213	4319485	90						
**11**	+	4319621	4320052	143	terminase, small subunit		pfam05119 (2.3E-21)	Terminase small subunit [*Enterobacteria* phage HK97]	99.8	1.2E-17

**12**	+	4320015	4321835	606	terminase, large subunit	gi|168495132|ref|YP_001686870.1| (4.2E-177)	COG4626 (1.4E-58)	Putative terminase large subunit [*Shigella* phage SfV]	100	3.5E-60
**13**	+	4321850	4323835	661	major capsid/prohead protease, HK97 family	lcl|209552422-3| (2.2E-168)	TIGR01554 (1.1E-19), TIGR01543 (7.8E-09)	Capsid polyprotein [*Pseudomonas* phage PAJU2]	100	1.5E-60
								Major capsid protein Rcc01687, g5 [*Rhodobacter capsulatus* DE442]	99.9	1.5E-24
**14**	+	4323891	4324190	99						
**15**	+	4324197	4325561	454	portal protein	gi|157166043|ref|YP_001449295.1| (1.1E-156)	pfam04860 (1.2E-82)	portal protein [*Streptomyces* phage phiC31]	100	3.3E-42
								Phage portal protein Rcc01684, HK97 family, g3 [*Rhodobacter capsulatus*]	100	1.7E-41
**16**	+	4325554	4327500	648	LPXTG-anchored collagen-like adhesin Scl2/SclB	gi|20065816|ref|NP_612899.1| (2.7E-21)	NF038329 (4.2E-29)			
**17**	+	4327506	4327790	94	adaptor protein	gi|209552427|ref|YP_002284342.1| (9.3E-09)	cd08054 (4.7E-20)	Head-tail connector protein [*Escherichia* virus HK97]	99.7	2.3E-15
								Adaptor protein Rcc01688, g6 [*Rhodobacter capsulatus*]	99.3	1.7E-10
**18**	+	4327787	4328152	121	stopper protein		pfam05521 (2.1E-14)	Stopper protein Rcc01689, g7 [*Rhodobacter capsulatus*]	99.8	6.9E-20
**19**	+	4328297	4328794	165	tail completion protein	gi|195546542|ref|YP_002117570.1| (6.9E-14)	pfam04883 (2.9E-06)	Capsid assembly protein G [*Escherichia* phage Mu]	98.8	1.6E-08
								Tail completion protein S [*Escherichia* phage P2]	97.5	9.7E-04

								Tail completion protein Z [*Escherichia* phage lambda]	93.3	0.9
**20**	+	4328791	4329189	132	tail terminator protein	gi|195546543|ref|YP_002117571.1| (3.5E-16)	pfam11367 (7.0E-25)	Tail terminator protein Rcc01690, g8 [*Rhodobacter capsulatus*]	99.9	4.5E-21
**21**	+	4329211	4329657	148	tail tube protein		pfam06199 (1.3E-21)	Phage major tail protein, TP901-1 family, Rcc01691, g9 [*Rhodobacter capsulatus*]	99.8	3.6E-18
**22**	+	4329657	4330067	136	tail tube protein, GTA-gp10		pfam11836 (2.8E-23)			
**23**	+	4330216	4333314	1032	tail tape measure protein	lcl|45686322-2| (7.1E-18)	pfam06791 (6.7E-32)	Tape measure protein [*Escherichia* phage lambda]	100	8.8E-25
**24**	+	4333314	4333949	211	distal tail protein		pfam09343 (1.3E-104)	Baseplate of native GTA particle, Rcc01695, g12 [*Rhodobacter capsulatus*]	100	3.9E-49
**25**	+	4333946	4334830	294	hub	gi|331028136|ref|YP_004421850.1| (6.7E-83)	TIGR02218 (1.3E-74), pfam09931 (1.1E-73)	Baseplate of native GTA particle, Rcc01696, g13 [*Rhodobacter capsulatus*]	100	6.1E-55
**26**	+	4334811	4335263	150	peptidase		TIGR02219 (3.8E-51)			
**27**	+	4335260	4339165	1301	megatron		pfam13547 (0); pfam13550 (1.6E-41)	Baseplate of native GTA particle, Rcc01698, g15 [*Rhodobacter capsulatus*]	100	3.2E-201
28	+	4339158	4340825	555	tail fiber protein	lcl|331028139-1| (6.0E-19)	pfam10983 (1.6E-31), cd10144 (3.6E-31)	L-shaped tail fiber protein [*Escherichia* phage T5]	99.6	3.6E-13
29	+	4340857	4341498	213	lysozyme	lcl|167832390-1| (2.3E-16)	COG3926 (9.8E-61)			
30	+	4341600	4342166	188		gi|160700617|ref|YP_001552292.1| (1.1E-19)				

31	+	4342170	4342577	135	spanin, inner membrane subunit		Spanin, inner membrane subunit [*Escherichia* phage P2]	96.6	0.3
32	+	4342574	4342846	90					
33	-	4343186	4343914	242	SDR family oxidoreductase	PRK06500 (9.8E-78)			
34	+	4344053	4344733	226					
35	+	4344993	4346051	352	methyltransferase FkbM family	TIGR01444 (7.4E-28)			
36	-	4346011	4346343	110	IS630 family transposase	NF033545 (3.1e-04)			
37	+	4346718	4346996	92	two component system sensor kinase	PRK15347 (8.8E-3)			
38	+	4346975	4348768	597	glycosyltransferase ExpE7 and similar proteins	cd03823 (4.0E-20)			
39	+	4348765	4350690	641	glycosyltransferase family A (GT-A)	cd06433 (3.7E-30)			
**LC4-associated prophage remnants**									
1	-	6635906	6636895	329	catalase-like heme-binding proteins	cd08153 (3.4E-139)			
2	+	6637170	6638150	326	regulator of protease activity HflC	COG0330 (1.3E-65)			
3	+	6638157	6638591	144	membrane protein in regulation of membrane protease activity	COG1585 (2.0E-27)			
4	-	6638624	6639409	261	nucleoside triphosphate pyrophosphohydrolase (MazG)	PRK09562 (5.8E-129)			

5	+	6640257	6641336	359	plasmid encoded RepA protein		pfam04796 (2.3E-27)			
6	+	6641450	6641887	145	the vicinal oxygen chelate (VOC) superfamily protein		cd07251 (1.4E-49)			
7	+	6641990	6642487	165	restriction endonuclease		pfam05066 (9.7E-10)			
8	-	6642497	6643234	245	LexA repressor	gi|323481895|ref|ADX81330.1| (9.7E-07)	cd06462 (5.7E-06)			
9	+	6643472	6643624	50	protocadherin			Protocadherin-15 [*Mus musculus*]	90.4	2.8
10	+	6643689	6644345	218	transcription termination/antitermination factor NusG		TIGR00922 (7.4E-22)			
**11**	+	6644761	6645333	190	terminase, small subunit			Terminase small subunit [*Lactococcus* phage SK1]	95.5	5.1E-02
**12**	+	6645251	6646735	494	terminase, large subunit	gi|9630534|ref|NP_046964.1| (1.4E-39)	COG5410 (1.5E-45)			
**13**	+	6646735	6648000	421	portal protein	gi|197085633|ref|YP_002128453.1| (2.7E-62)	pfam06381 (2.7E-110)	Portal protein [*Vibrio* phage XM1]	100	2.6E-40
								Phage portal protein Rcc01684, HK97 family, g3 [*Rhodobacter capsulatus*]	99.9	2.4E-24
**14**	+	6648373	6649365	330	prohead protease	lcl|197085641-1| (7.1E-36)	pfam09979 (7.5E-66)	Prohead core protein protease [*Enterobacteria* phage T4]	94.9	0.9
								Prohead protease [*Enterobacteria* phage HK97]	92.6	2

**15**	+	6649365	6649802	145	Capsid fiber protein			Minor capsid protein [*Vibrio* phage XM1]	98.6	1.4E-06
								Capsid fiber protein [*Bacillus* phage phi29]	98.4	1.8E-05
**16**	+	6649814	6650761	315	major capsid protein	gi|161617968|ref|YP_001595448.1| (4.8E-33)	pfam09950 (1.6E-112)	Major capsid protein [*Vibrio* phage XM1]	100	8.3E-44
								Major capsid protein Rcc01687, g5 [*Rhodobacter capsulatus* DE442]	98.9	2.0E-07
**17**	+	6650764	6650973	69						
**18**	+	6650976	6651341	121	adaptor		pfam13262 (1.1E-09)	Head completion protein [*Vibrio* phage XM1]	100.0	2.9E-31
								Head completion protein gp15 [*Bacillus* phage SPP1]	92.1	1.2
**19**	+	6651341	6651664	107	stopper			Head-tail connector protein FII [*Escherichia* phage lambda]	98.9	1.0E-07
**20**	+	6651664	6651930	88						
**21**	+	6651932	6652345	137	tail terminator protein		pfam11367 (3.8E-18)	Tail terminator protein Rcc01690, g8 [*Rhodobacter capsulatus*]	99.9	2.8E-22
**22**	+	6652439	6652879	146	tail tube protein		pfam06199 (1.7E-20)	Phage major tail protein, TP901-1 family, Rcc01691, g9 [*Rhodobacter capsulatus*]	99.9	8.8E-19
**23**	+	6652888	6653469	193	tail tube protein, GTA-gp10		pfam11836 (5.7E-16)	Tail assembly protein E [*Escherichia* phage P2]	97.2	4.4E-02

**24**	+	6653453	6654478	341	tail tape measure protein			Tape measure protein [*Escherichia* phage T1]	99.1	3.1E-07
								Tape measure protein [*Escherichia* phage N15]	98.6	6.4E-05
**25**	+	6654480	6655115	211	distal tail protein		pfam09343 (2.7E-104)	Baseplate of native GTA particle, Rcc01695, g12 [*Rhodobacter capsulatus*]	100	1.7E-48
**26**	+	6655112	6655996	294	hub	gi|331028136|ref|YP_004421850.1| (6.8E-81)	pfam09931 (2.0E-73), pfam09356 (2.2E-37)	Baseplate of native GTA particle, Rcc01696, g13 [*Rhodobacter capsulatus*]	100	2.5E-53
**27**	+	6655977	6656429	150	peptidase		TIGR02219 (2.0E-61)			
**28**	+	6656426	6660328	1300	megatron		pfam13547 (0), pfam13550 (6.1E-39)	Baseplate of native GTA particle, Rcc01698, g15 [*Rhodobacter capsulatus*]	100	6.8E-206
29	+	6660321	6661988	555	tail fiber protein	lcl|331028139-1| (7.8E-19)	pfam10983 (6.1E-34), cd10144 (4.1E-31)	L-shaped tail fiber protein [*Escherichia* phage T5]	99.6	8.1E-14
30	+	6662063	6662638	191	lysozyme	lcl|331028139-1| (7.8E-19)	COG3926 (1.7E-53)			
31	+	6662689	6662877	62						
32	-	6663127	6663951	274	lipoprotein-anchoring transpeptidase ErfK/SrfK		COG1376 (1.8E-48)			
33	+	6664247	6664747	166	ROS/MUCR transcriptional regulator protein		pfam05443 (1.8E-75)			
34	+	6664855	6665151	98				Uncharacterized protein 6 [*Halorubrum pleomorphic* virus 1]	91.2	0.9

35	-	6665403	6666293	296	exonuclease RNase T and DNA polymerase III	gi|347449610|ref|AEO93651.1| (1.1E-09)	PRK09182 (9.8E-135)
36	-	6666385	6667686	433	Shikimate transporter, major facilitator superfamily		cd17369 (5.3E-88)
37	-	6667781	6669334	517	D-galactarate dehydratase / Altronate hydrolase		pfam04295 (0)
38	-	6669374	6670111	245	ABC-type branched-chain amino acid transport system, ATPase component (LivF)		COG0410 (1.1E-114), cd03224 (4.9E-111)
39	-	6670211	6672046	611	ABC-type branched-chain amino acid transport system, ATPase component (LivG)		COG0411 (3.1E-102)
40	-	6672043	6672918	291	ABC-type branched-chain amino acid transport system, permease component (LivH)		cd06582 (6.3E-73), COG0559 (1.5E-57)

[a]ORFs of LC1–LC5 are indicated in bold.

[b]Putative functions are predicted based on the function of homologs from the Conserved Domain Database or detected by HHpred search against the PDB, SCOPe, and UniprotKb/Swiss-Prot databases.

[c]Top hit of each ORF in the Phage Orthologous Group (POG) database and its e-value.

[d]Significant hits of each ORF in the Conserved Domain Database and their e-values.

[e]HHpred search against the PDB, SCOPe, and UniprotKb/Swiss-Prot databases were conducted on ORFs without conserved domain or in the genomic regions mainly encoding structural proteins.

[f]Homologs belonging to the RcGTA proteins were indicated in purple.

Table S5. *Methylobacterium nodulans* ORS 2060 LC1–LC5 genes homologous to each other LC genes and to the RcGTA-like cluster genes.

ORF	Strand	NR accession no.	aa length	Putative function	RcGTA-like homologs	LC1	LC2	LC3	LC4	LC5	RcGTA	Top hit of the other RcGTA-like gene cluster (aa identity (%))	Order of the top hit bacterial strain
**LC1**													
27	+	ACL56980.1	144	terminase small subunit		-	46.8	46.8					
28	+	ACL56981.1	611	terminase large subunit		-	83.9	83.6		25.6			
30	+	ACL56983.1	473	portal protein	g3	-				33.0		*Parvularcula bermudensis* HTCC2503 (28.2)	*Parvularculales*
32	+	ACL56985.1	438	major capsid protein	g5	-	23.1	23.1		24.7		*Novosphingobium* sp. PP1Y (26.2)	*Sphingomonadales*
35	+	ACL56988.1	162	adaptor	g6	-				30.3		*Parvibaculum lavamentivorans* DS-1 (28.4)	*Rhizobiales*
36	+	ACL56989.1	109	stopper		-				38.9			
37	+	ACL56990.1	190	tail completion protein		-				35.5			
38	+	ACL56991.1	149	tail terminator protein		-				39.6			
39	+	ACL56992.1	149	tail tube protein		-	27.0	27.0		44.3			
40	+	ACL56993.1	128	tail assembly protein, GTA-gp10		-	36.8	36.8		59.2			
42	+	ACL56995.1	735	tail tape measure protein	g11	-	84.3	76.4	45.7	31.5		*Methylocystis* sp. SC2 (35.4)	*Rhizobiales*
43	+	ACL56996.1	211	distal tail	g12	-	98.6	95.7	95.7	97.6	55.1	*Rhodobacter sphaeroides* ATCC 17025 (61.4)	*Rhodobacterales*
44	+	ACL56997.1	294	hub	g13	-	95.6	94.2	100	99.7	45.4	*Hyphomicrobium* sp. MC1 (49.3)	*Rhizobiales*
45	+	ACL56998.1	150	peptidase	g14	-	94	94.7	99.3	99.3	48.6	*Oligotropha carboxidovorans* OM5 (58.6)	*Rhizobiales*
46	+	ACL56999.1	1300	megatron	g15	-	95.2	96.2	99.8	99.3	45.9	*Hyphomicrobium nitrativorans* NL23 (47.0)	*Rhizobiales*

47	+	ACL57000.1	555	tail fiber protein	*rcc00171*	-	91.0	94.2	98.6	98.6	46.6	*Methylorubrum extorquens* DM4 (74.3)	*Rhizobiales*
48	+	ACL57001.1	213	lysozyme	*rcc00555*	-	91.5	87.3	67.3	69.8	44.5	*Pelagibacterium halotolerans* B2 (50.6)	*Rhizobiales*
**LC2**													
1	-	ACL57560.1	213	lysozyme	*rcc00555*	91.5	-	85.0	66.9	69.3	43.7	*Pelagibacterium halotolerans* B2 (51.4)	*Rhizobiales*
2	-	ACL57561.1	554	tail fiber protein	*rcc00171*	91.0	-	93.0	91.3	91.3	45.2	*Methylorubrum extorquens* DM4 (70.2)	*Rhizobiales*
3	-	ACL57562.1	1301	megatron	g15	95.2	-	95.0	95.0	94.9	45.8	*Hyphomicrobium nitrativorans* NL23 (47.0)	*Rhizobiales*
4	-	ACL57563.1	150	peptidase	g14	94	-	94	94.7	94.7	48.3	*Oligotropha carboxidovorans* OM5-ATCC49405 (58.3)	*Rhizobiales*
5	-	ACL57564.1	294	hub	g13	95.6	-	93.2	95.6	95.9	45.4	*Rhodopseudomonas palustris* CGA009 (50.7)	*Rhizobiales*
6	-	ACL57565.1	211	distal tail	g12	98.6	-	95.3	95.3	96.2	54.1	*Rhodobacter sphaeroides* ATCC 17025 (61.8)	*Rhodobacterales*
7	-	ACL57566.1	1032	tail tape measure protein	g11	97.6	-	95.1	41.9	40.3		*Leisingera methylohalidivorans* DSM 14336 (34.5)	*Rhodobacterales*
8	-	ACL57567.1	136	tail tube protein, GTA-gp10		36.8	-	100	36.9	34.6			
9	-	ACL57568.1	148	tail tube protein	g9	27.0	-	99.3	55.1	24.8		*Methylobacterium populi* BJ001 (28.9)	*Rhizobiales*
10	-	ACL57569.1	132	tail terminator	g8		-	99.2				*Hyphomicrobium* sp. MC1 (36.4)	*Rhizobiales*
11	-	ACL57570.1	165	tail completion protein			-	93.3					
12	-	ACL57571.1	121	stopper			-	98.3					

13	-	ACL57572.1	94	adaptor			-	97.9				
14	-	ACL57573.1	767	LPXTG-anchored collagen-like adhesin Scl2/SclB			-	73.4				
15	-	ACL57574.1	454	portal protein	g3		-	100		26.1	*Phaeobacter inhibens* 2.10 (28.1)	*Rhodobacterales*
16	-	ACL57575.1	99				-	100				
17	-	ACL57576.1	661	major capsid protein, prohead protease, HK97 family	g5	23.1	-	100			*Agrobacterium vitis* S4 (29.8)	*Rhizobiales*
18	-	ACL57577.1	606	terminase large subunit		82.6	-	99.2	24.4			
19	-	ACL57578.1	143	terminase small subunit		46.8	-	98.6				
**LC3**												
11	+	ACL59156.1	143	terminase small subunit		46.8	98.6	-				
12	+	ACL59157.1	606	terminase large subunit		83.6	99.2	-	24.4			
13	+	ACL59158.1	661	major capsid protein, prohead protease, HK97 family	g5	23.1	100	-			*Agrobacterium vitis* S4 (29.8)	*Rhizobiales*
14	+	ACL59159.1	99				100	-				
15	+	ACL59160.1	454	portal protein	g3	24.2	100	-	24.4	26.1	*Phaeobacter inhibens* 2.10 (28.1)	*Rhodobacterales*
16	+	ACL59161.1	648	LPXTG-anchored collagen-like adhesin Scl2/SclB			67.3	-				
17	+	ACL59162.1	94	adaptor			97.9	-				
18	+	ACL59163.1	121	stopper			98.3	-				
19	+	ACL59164.1	165	tail completion protein			93.3	-				
20	+	ACL59165.1	132	tail terminator	g8		99.2	-			*Hyphomicrobium* sp. MC1 (36.4)	*Rhizobiales*

21	+	ACL59166.1	148	tail tube protein	g9	27.0	99.3	-	55.8	25.5		*Methylobacterium populi* BJ001 (28.4)	*Rhizobiales*
22	+	ACL59167.1	136	tail assembly protein, GTA-gp10		36.8	100	-	36.9	34.6			
23	+	ACL59168.1	1032	tail tape measure protein	g11	97.4	95.1	-	44.2	40.2		*Streptomyces purpurogeneiscleroticus* strain NRRL B-2952 (31.6)	*Streptomycetales*
24	+	ACL59169.1	211	distal tail	g12	95.7	95.3	-	96.2	98.1	55.1	*Rhodobacter sphaeroides* ATCC 17025 (61.8)	*Rhodobacterales*
25	+	ACL59170.1	294	hub	g13	94.2	93.2	-	94.2	93.9	46.4	*Nitrobacter winogradskyi* Nb-255 (52.2)	*Rhizobiales*
26	+	ACL59171.1	150	peptidase	g14	94.7	94	-	95.3	95.3	47.9	*Oligotropha carboxidovorans* OM5-ATCC49405 (58.3)	*Rhizobiales*
27	+	ACL59172.1	1301	megatron	g15	96.2	95.0	-	96.4	96.1	46.1	*Hyphomicrobium nitrativorans* NL23 (47.3)	*Rhizobiales*
28	+	ACL59173.1	555	tail fiber protein	*rcc00171*	94.2	93.0	-	94.9	94.9	45.7	*Methylorubrum extorquens* DM4 (69.7)	*Rhizobiales*
29	+	ACL59174.1	213	lysozyme	*rcc00555*	87.3	85.0	-	68.7	71.2	44.9	*Pelagibacterium halotolerans* B2 (50)	*Rhizobiales*
**LC4**													
18	+	ACL61336.1	137	tail terminator protein	g8				-			*Hyphomicrobium denitrificans* ATCC 51888 (31.0)	*Rhizobiales*
19	+	ACL61337.1	146	tail tube protein	g9		55.1	55.8	-	28.8		*Methylobacterium populi* BJ001 (33.6)	*Rhizobiales*
20	+	ACL61338.1	193	tail assembly protein, GTA-gp10			36.9	36.9	-	33.3			
21	+	ACL61339.1	341	tail tape measure protein	g11	43.7	40.6	43.2	-	42.9		*Erythrobacter litoralis* HTCC2594 (39.5)	*Sphingomonadales*

22	+	ACL61340.1	211	distal tail	g12	95.7	95.3	96.2	-	98.1	55.3	*Rhodobacter sphaeroides* ATCC 17025 (59.6)	*Rhodobacterales*
23	+	ACL61341.1	294	hub	g13	100	95.6	94.2	-	99.7	45.4	*Hyphomicrobium* sp. MC1 (49.3)	*Rhizobiales*
24	+	ACL61342.1	150	peptidase	g14	99.3	94.7	95.3	-	100	47.9	*Oligotropha carboxidovorans* OM5 (59.3)	*Rhizobiales*
25	+	ACL61343.1	1300	megagtron	g15	99.8	95.0	96.4	-	99.5	45.9	*Hyphomicrobium nitrativorans* NL23 (47.0)	*Rhizobiales*
26	+	ACL61344.1	555	tail fiber protein	*rcc00171*				-		46.6	*Methylorubrum extorquens* DM4 (74.3)	*Rhizobiales*
27	+	ACL61345.1	191	lysozyme	*rcc00555*	62.8	66.3	64.5	-	95.3	37.8	*Pelagibacterium halotolerans* B2 (46.4)	*Rhizobiales*
**LC5**													
1	-	ACL62049.1	191	lysozyme	*rcc00555*	65.0	68.6	66.7	95.3	-	40.3	*Pelagibacterium halotolerans* B2 (45.4)	*Rhizobiales*
2	-	ACL62050.1	555	tail fiber protein	*rcc00171*	98.6	91.3	94.9	100	-	46.6	*Methylorubrum extorquens* DM4 (74.3)	*Rhizobiales*
3	-	ACL62051.1	1300	megatron	g15	99.3	94.9	96.1	99.5	-	45.8	*Hyphomicrobium nitrativorans* NL23 (47.1)	*Rhizobiales*
4	-	ACL62052.1	150	peptidase	g14	99.3	94.7	95.3	100	-	47.9	*Oligotropha carboxidovorans* OM5 (59.3)	*Rhizobiales*
5	-	ACL62053.1	294	hub	g13	99.7	95.9	93.9	99.7	-	45.4	*Hyphomicrobium* sp. MC1 (49.3)	*Rhizobiales*
6	-	ACL62054.1	211	distal	g12	97.6	96.2	98.1	98.1	-	55.1	*Methylocystis* sp. SC2 (59.9)	*Rhizobiales*
7	-	ACL62055.1	674	tail tape measure protein	g11	30.6	40.3	40.2	37.9	-	26.8	*Brucella suis* ATCC 23445 (32.7)	*Rhizobiales*

9	-	ACL62057.1	131	tail tube protein, GTA-gp10		59.2	34.6	34.6		-			
10	-	ACL62058.1	149	tail tube protein		44.3	24.8	25.5	28.8	-			
12	-	ACL62060.1	136	tail terminator protein		39.6				-			
13	-	ACL62061.1	184	tail completion protein		35.5				-			
14	-	ACL62062.1	112	stopper		38.9				-			
18	-	ACL62066.1	201	adaptor	g6	30.3				-		*Parvibaculum lavamentivorans* DS-1 (34.7)	*Rhizobiales*
20	-	ACL62068.1	417	major capsid protein	g5	24.7				-	28.6	*Ruegeria pomeroyi* DSS3 (31.7)	*Rhodobacterales*
22	-	ACL62070.1	428	portal protein	g3	33.0	24.4	24.4		-	28.9	*Rhodopseudomonas palustris* CGA009 (29.3)	*Rhizobiales*
23	-	ACL62071.1	581	terminase large subunit		25.6	28.1	24.4		-			

Table S6. Genomes containing RcGTA-like head-tail clusters used in this study.

Organism[a]	Order	GenBank accession no.
**Alphaproteobacteria**		
Caulobacter crescentus NA1000	*Caulobacterales*	CP001340.1
Caulobacter segnis ATCC 21756	*Caulobacterales*	CP002008.1
Caulobacter sp. K31	*Caulobacterales*	CP000927.1
Hirschia baltica ATCC 49814	*Hyphomonadales*	CP001678.1
Maricaulis maris MCS10	*Maricaulales*	CP000449.1
Parvularcula bermudensis HTCC2503	*Parvularculales*	CP002156.1
Agrobacterium fabrum str. C58	*Rhizobiales*	AE007869.2
Agrobacterium fabrum str.1D132	*Rhizobiales*	CP033022.1
Agrobacterium sp. H13-3	*Rhizobiales*	CP002248.1
Agrobacterium vitis S4	*Rhizobiales*	CP000633.1
Azorhizobium caulinodans ORS 571	*Rhizobiales*	AP009384.1
Brucella abortus A13334	*Rhizobiales*	CP003176.1
Brucella abortus bv. 1 str. 9-941	*Rhizobiales*	AE017223.1
Brucella abortus S19	*Rhizobiales*	CP000887.1
Brucella canis ATCC 23365	*Rhizobiales*	CP000872.1
Brucella canis HSK A52141	*Rhizobiales*	CP003174.1
Brucella melitensis ATCC 23457	*Rhizobiales*	CP001488.1
Brucella melitensis biovar Abortus 2308	*Rhizobiales*	AM040264.1
Brucella melitensis bv. 1 str. 16M	*Rhizobiales*	AE008917.1
Brucella melitensis M28	*Rhizobiales*	CP002459.1
Brucella melitensis M5-90	*Rhizobiales*	CP001851.1
Brucella melitensis NI	*Rhizobiales*	CP002931.1
Brucella microti CCM 4915	*Rhizobiales*	CP001578.1
Brucella ovis ATCC 25840	*Rhizobiales*	CP000708.1
Brucella pinnipedialis B2-94	*Rhizobiales*	CP002078.1
Brucella suis 1330	*Rhizobiales*	AE014291.4
Brucella suis 1330-2	*Rhizobiales*	CP002997.1
Brucella suis ATCC 23445	*Rhizobiales*	CP000911.1
Brucella suis VBI22	*Rhizobiales*	CP003128.1
Hyphomicrobium denitrificans 1NES1	*Rhizobiales*	CP005587.1
Hyphomicrobium denitrificans ATCC 51888	*Rhizobiales*	CP002083.1
Hyphomicrobium nitrativorans NL23	*Rhizobiales*	CP006912.1
Hyphomicrobium sp. MC1	*Rhizobiales*	FQ859181.1
Methylorubrum extorquens AM1	*Rhizobiales*	CP001510.1
Methylorubrum extorquens CM4	*Rhizobiales*	CP001298.1
Methylorubrum extorquens DM4	*Rhizobiales*	FP103042.2
Methylorubrum extorquens PA1	*Rhizobiales*	CP000908.1
Methylorubrum populi BJ001	*Rhizobiales*	CP001029.1
Methylobacterium radiotolerans JCM 2831	*Rhizobiales*	CP001001.1
Methylocystis sp. SC2	*Rhizobiales*	HE956757.1

Nitrobacter hamburgensis X14	*Rhizobiales*	CP000319.1
Nitrobacter winogradskyi Nb-255	*Rhizobiales*	CP000115.1
Oligotropha carboxidovorans OM4	*Rhizobiales*	CP002821.1
Oligotropha carboxidovorans OM5-ATCC49405	*Rhizobiales*	CP001196.1
Oligotropha carboxidovorans OM5	*Rhizobiales*	CP002826.1
Parvibaculum lavamentivorans DS-1	*Rhizobiales*	CP000774.1
Pelagibacterium halotolerans B2	*Rhizobiales*	CP003075.1
Pseudovibrio sp. FOBEG1	*Rhizobiales*	CP003147.1
Rhizobium sp. IRBG74	*Rhizobiales*	HG518322.1
Rhodopseudomonas palustris BisA53	*Rhizobiales*	CP000463.1
Rhodopseudomonas palustris BisB18	*Rhizobiales*	CP000301.1
Rhodopseudomonas palustris BisB5	*Rhizobiales*	CP000283.1
Rhodopseudomonas palustris CGA009	*Rhizobiales*	BX572599.1
Rhodopseudomonas palustris DX-1	*Rhizobiales*	CP002418.1
Rhodopseudomonas palustris HaA2	*Rhizobiales*	CP000250.1
Rhodopseudomonas palustris TIE-1	*Rhizobiales*	CP001096.1
Starkeya novella DSM 506	*Rhizobiales*	CP002026.1
Xanthobacter autotrophicus Py2	*Rhizobiales*	CP000781.1
Dinoroseobacter shibae DFL 12	*Rhodobacterales*	CP000830.1
Jannaschia sp. CCS1	*Rhodobacterales*	CP000264.1
Ketogulonicigenium vulgare WSH-001	*Rhodobacterales*	CP002018.1
Ketogulonicigenium vulgare Y25	*Rhodobacterales*	CP002224.1
Leisingera methylohalidivorans DSM 14336	*Rhodobacterales*	CP006773.1
Oceanicola granulosus HTCC2516	*Rhodobacterales*	NZ_CH724107.1
Octadecabacter antarcticus 307	*Rhodobacterales*	CP003740.1
Octadecabacter arcticus 238	*Rhodobacterales*	CP003742.1
Paracoccus aminophilus JCM 7686	*Rhodobacterales*	CP006650.1
Paracoccus denitrificans PD1222	*Rhodobacterales*	CP000490.1
Phaeobacter gallaeciensis 2.10	*Rhodobacterales*	CP002972.1
Phaeobacter gallaeciensis DSM 26640	*Rhodobacterales*	CP006966.1
Phaeobacter inhibens DSM 17395	*Rhodobacterales*	CP002976.1
Polymorphum gilvum SL003B-26A1	*Rhodobacterales*	CP002568.1
Pseudooceanicola batsensis HTCC2597	*Rhodobacterales*	NZ_CH724131.1
Rhodobacter capsulatus SB 1003	*Rhodobacterales*	CP001312.1
Rhodobacter sphaeroides 2.4.1	*Rhodobacterales*	CP000143.2
Rhodobacter sphaeroides ATCC 17025	*Rhodobacterales*	CP000661.1
Rhodobacter sphaeroides ATCC 17029	*Rhodobacterales*	CP000577.1
Rhodobacter sphaeroides KD131	*Rhodobacterales*	CP001150.1
Roseobacter denitrificans OCh114	*Rhodobacterales*	CP000362.1
Roseobacter litoralis OCh149	*Rhodobacterales*	CP002623.1
Ruegeria pomeroyi DSS-3	*Rhodobacterales*	CP000031.2
Ruegeria sp. TM1040	*Rhodobacterales*	CP000377.1
Roseobacter sp. MED193	*Rhodobacterales*	AANB01000008.1
Roseovarius nubinhibens ISM	*Rhodobacterales*	AALY01000001.1

Sulfitobacter sp. EE-36	*Rhodobacterales*	AALV01000003.1
Yoonia vestfoldensis SMR4r	*Rhodobacterales*	CP021431.1
Erythrobacter litoralis HTCC2594	*Sphingomonadales*	CP000157.1
Erythrobacter sp. NAP1	*Sphingomonadales*	AAMW01000002.1
Novosphingobium aromaticivorans DSM 12444	*Sphingomonadales*	CP000248.1
Novosphingobium sp. PP1Y	*Sphingomonadales*	FR856862.1
Sphingobium chlorophenolicum L-1	*Sphingomonadales*	CP002798.1
Sphingobium japonicum UT26S	*Sphingomonadales*	AP010803.1
Sphingobium sp. SYK-6	*Sphingomonadales*	AP012222.1
Sphingomonas sp. MM-1	*Sphingomonadales*	CP004036.1
Sphingopyxis alaskensis RB2256	*Sphingomonadales*	CP000356.1
**Gammaproteobacteria**		
*Pseudomonas_bauzanensis*_W13Z2	*Pseudomonadales*	JFHS01000006.1
**Actinobacteria**		
Streptomyces purpurogeneiscleroticus NRRL B-2952	*Streptomycetales*	LGEI01000102.1

[a]Nine alphaproteobacterial strains additional to those included in Shakya et al. (2) were indicated in blue.

REFERENCES

1. Kristensen DM, Waller AS, Yamada T, Bork P, Mushegian AR, Koonin EV. 2013. Orthologous gene clusters and taxon signature genes for viruses of prokaryotes. J Bacteriol 195:941–950.

2. Shakya M, Soucy SM, Zhaxybayeva O. 2017. Insights into origin and evolution of α-proteobacterial gene transfer agents. Virus Evol 3:vex036.
